# Correction: Global learning opportunities within social innovation in health (GLOWS): A modified Delphi process to identify and pilot core competencies for learning

**DOI:** 10.1371/journal.pone.0352087

**Published:** 2026-06-17

**Authors:** Emily Wallace, Yusha Tao, Ogechukwu B. Aribodor, Zixuan Zhu, Angelica Borbón, Beatrice Halpaap, Bertha M. Chakhame, Eunice C. Jacob, Fatema Ahmed, Joel Msafiri Francis, Komang G. Septiawan, Kovey Mawuli, Linet Mutisya, Marlita Putri Ekasari, Nwadiuto Okwuniru Azugo, Tina Fourie, Adriana S. Ruiz, Jackeline Alger, Abigail Ruth Mier, Weiming Tang, Gloria Aidoo-Frimpong, Jackline Nanono, Jesson James A. Montealto, Obidimma Ezezika, Per Kåks, Wenjie Shan, Jana Deborah Mier-Alpano, Gifty Marley, Elizabeth Chen, Joseph D. Tucker

The affiliation for the 27th author is incorrect. Jana Deborah Mier-Alpano is not affiliated with #18 but with #20: Social Innovation in Health Initiative - Philippines Hub, Manila, Philippines.
