# Supplementary material for: Global learning opportunities within social innovation in health (GLOWS): A modified Delphi process to identify and pilot core competencies for learning
Source: PLoS One. 2026 Jan 9;21(1):e0339359. doi: 10.1371/journal.pone.0339359 (PMC12788671; doi:10.1371/journal.pone.0339359)
Supplement: S1 Table — (DOCX) [file pone.0339359.s001.docx]

Supplementary Table 1: Description of each session of the Social Innovation in Health Mid-year Training Workshop

| **Title** | | **Summary** | **Learning Competencies** |
| --- | --- | --- | --- |
| Week 1 | Introduction to Participatory Health Research Methods, Emphasizing Designathons | Participatory research approaches like community advisory boards, co-creation, and design-a-thons provide an avenue for formative assessments and serve as the basis for collaboration with end-users in identifying solutions to gaps in implementation and improving efficiency. | - Community Engagement - User-centred design - Crowd-sourcing |
| Week 2 | Social Innovation in Health: Introduction to Social Innovation Theory and Knowledge, Attitudes, Practices, and Behaviours (KAP-B) Framework | This session introduces the audience to the concepts and applications of social innovation theories in health. The learning objectives of this session include: A. To define social innovation in health and its implications for inclusivity and community engagement. B. To explain social innovation theories, such as diffusion of Innovations Theory and Social Capital Theory, and their relevance in creating social value and systemic change. C. To explain the KAP-B Framework and its application in addressing social issues. | - Community Engagement - Theories and Frameworks |
| Week 3 | Introduction to Human-Centered Design for Health | This session focuses on the principles of human-centered design and its application through an online interactive workshop. The session will briefly cover basic concepts, such as the phases of human-centered design, before guiding participants in a series of activities to apply what they have learned to a case vignette provided. This session aims to equip participants with knowledge, tools, and resources related to human-centered design that they can use for their work in health and social innovation. | - Human Centred Design - Empathy |
| Week 4 | Applying an Intersectional Gender Lens on Social Innovations in Health: An Introductory Module | We are all shaped by different aspects of identity, such as race, gender, and social class. Intersectionality helps us understand how these layers interact and influence our experiences. This session will focus on gender as a key entry point into intersectional analysis, examining how gender power relations intersect with other social factors to impact lives and create diverse needs and experiences. Through this one-hour interactive session, participants will gain an overview of the importance of gender frameworks in research and learn how to apply an intersectional gender framework in social innovations in health. Understanding these intersections is essential for future gender-sensitive and transformative research. | - Intersectionality - Social Determinants of Health - Navigating Diverse Cultural Contexts - Health Disparities |
| Week 5 | Co-Creation: An Innovative Communal Approach in Addressing Social Health Challenges | This workshop session is designed for individuals and groups interested in using a Living Lab Bayanihan- a communal approach focused on co-creation and multi-stakeholder collaboration in addressing social health challenges. This session will include interactive activities and practical strategies for Problem Identification and the Importance of User/People and Stakeholder Engagement. | - Community Engagement |
| Week 6 | Democratizing Applied Behavioral and Decision Sciences (BeSci) to Combat MMR Vaccine Hesitancy through Behavioral Design Sprints | AHA! Behavioral Design® (AHA! BD) pioneers the local application of Behavioral Science (BeSci), offering a Global South interpretation. Integrating Behavioral and Cultural Insights (BCI) into health promotion expands BeSci beyond nudges to tackle complex public health and sustainability challenges. This led to the Behavioral Design Sprint (BeDesign Sprint™), turning otherwise technical BCI into practical and actionable health solutions co-created with the community themselves. The session showcases a case study of this approach and an interactive condensed simulation of AHA! BD's BeDesign Sprint™ for audiences to participate in. | - Human-centred Design |
| Week 7 | Embedding Social Innovation to Strengthen Health Systems and Leadership | This session explores how social innovation strengthens health systems by addressing current issues and developing key leadership competencies. It includes methodologies, tools, and success stories, such as the comprehensive intervention in La Guajira, Colombia, and the case of the Zika children. Leadership strategies that promote inclusion and collaboration among multiple stakeholders are highlighted. Additionally, participatory activities are conducted to prioritize health problems and analyze the perspectives from which leadership is exercised. The importance of social innovation in improving the efficiency and effectiveness of health interventions is emphasized, ensuring that the prioritized needs of the population are met. | - Leadership/team management - Sustainable innovations |
| Week 8 | From Idea to Impact: Mastering the Pitch for Social Innovations | You might have a groundbreaking social innovation, but it may not reach its full potential unless you convince others to believe in it through a compelling pitch. Participants will be introduced to key elements and strategies for an excellent pitch to effectively communicate a social innovation with a broad range of communities, especially people with lived experience and potential partners. In addition, through case studies, you will be exposed to concepts that can enhance your entrepreneurial skills to raise funds for social innovation and innovative financing. | - Pitching - Storytelling - Fund Raising |
