# Supplementary material for: Global learning opportunities within social innovation in health (GLOWS): A modified Delphi process to identify and pilot core competencies for learning
Source: PLoS One. 2026 Jan 9;21(1):e0339359. doi: 10.1371/journal.pone.0339359 (PMC12788671; doi:10.1371/journal.pone.0339359)
Supplement: S3 File — (DOCX) [file pone.0339359.s003.docx]

**S4: Survey One**

[**GLOWS: Global Learning Opportunities Within Social Innovation (jotform.com)**](https://form.jotform.com/241242842128047)
