# Supplementary material for: Global learning opportunities within social innovation in health (GLOWS): A modified Delphi process to identify and pilot core competencies for learning
Source: PLoS One. 2026 Jan 9;21(1):e0339359. doi: 10.1371/journal.pone.0339359 (PMC12788671; doi:10.1371/journal.pone.0339359)
Supplement: S4 File — (DOCX) [file pone.0339359.s004.docx]

**S5: Survey Two Link**

[**Survey 2: Global Learning Opportunities Within Social Innovation (jotform.com)**](https://form.jotform.com/241745028024349)
