# Supplementary material for: Global learning opportunities within social innovation in health (GLOWS): A modified Delphi process to identify and pilot core competencies for learning
Source: PLoS One. 2026 Jan 9;21(1):e0339359. doi: 10.1371/journal.pone.0339359 (PMC12788671; doi:10.1371/journal.pone.0339359)
Supplement: S5 Table — (DOCX) [file pone.0339359.s005.docx]

S6 Table: Demographic characteristics of participants attending the Social Innovation Mid-year Training Workshop held between June 2024 and July 2024 (N=137).

| **Variable** | **Number (%)** | **Mean ± SD** |
| --- | --- | --- |
| **Age in years** | | 31 ± 9 |
| ≤30 | 75 (54.7%) |  |
| 31–50 | 54 (39.4%) |  |
| >50 | 6 (4.4%) |  |
| No reply | 2 (1.5%) |  |
| **WHO region** | | |
| Western Pacific Region | 69 (50.4%) |  |
| African Region | 35 (25.5%) |  |
| Region of the Americas | 18 (13.1%) |  |
| South-East Asian Region | 8 (5.8%) |  |
| European Region | 6 (4.4%) |  |
| Eastern Mediterranean Region | 1 (0.7%) |  |
| **Income region (from World Bank 2024)** | | |
| High income | 11 (8.0%) |  |
| Upper middle income | 82 (59.9%) |  |
| Lower middle income | 34 (24.8%) |  |
| Low income | 10 (7.3%) |  |
| **Gender** | | |
| Female | 96 (70.1%) |  |
| Male | 40 (29.2%) |  |
| Prefer not to say | 1 (0.7%) |  |
| **Racial or ethnic minority group** | | |
| Yes | 14 (10.2%) |  |
| No | 114 (83.2%) |  |
| Prefer not to say | 9 (6.6%) |  |
| **Associated with a SIHI hub** | | |
| Yes | 22 (16.1%) |  |
| No | 62 (45.3%) |  |
| No reply | 53 (38.7%) |  |
| **Currently a student (undergraduate, graduate, or postgraduate)** | | |
| Yes | 82 (59.9%) |  |
| No | 55 (40.1%) |  |
| **Highest educational degree** | | |
| PhD (or MD) | 26 (19.0%) |  |
| Master | 63 (46.0%) |  |
| Bachelor | 41 (29.9%) |  |
| High school | 7 (5.1%) |  |
| **The main field of training** | | |
| Public Health | 58 (42.3%) |  |
| Nursing | 41 (29.9%) |  |
| Medicine | 15 (10.9%) |  |
| Social Innovation and Social Science | 12 (8.8%) |  |
| Biological Sciences | 5 (3.6%) |  |
| Others | 6 (4.4%) |  |
| **Primary job duty** | | |
| Research | 25 (45.5%) |  |
| Teaching | 11 (20.0%) |  |
| Service | 4 (7.3%) |  |
| Clinician | 4 (7.3%) |  |
| Policy | 3 (5.5%) |  |
| Others | 8 (14.5%) |  |
| **Years of work** | | 9.0 ± 6.9 |
| ≤ 3 years | 15 (27.3%) |  |
| 4–7 years | 13 (23.6%) |  |
| 8-15 years | 15 (27.3%) |  |
| >15 | 11 (20.0%) |  |
| No reply | 1 (1.8%) |  |
| **Number of sessions attended** | | |
| One session | 1 (0.7%) |  |
| Two sessions | 7 (5.1%) |  |
| Three sessions | 0 (0.0%) |  |
| Four sessions | 4 (2.9%) |  |
| Five sessions | 7 (5.1%) |  |
| Six sessions | 21 (15.3%) |  |
| Seven sessions | 25 (18.2%) |  |
| Eight sessions | 72 (52.6%) |  |
| **Extent to which goals were met during the workshop** | | |
| Completely met | 35 (25.5%) |  |
| Mostly met | 72 (52.6%) |  |
| Slightly met | 3 (2.2%) |  |
| Partially met | 27 (19.7%) |  |
| Not met at all | 0 (0.0%) |  |
| **Overall appraisal of the training workshop series** | | |
| Excellent | 93 (67.9%) |  |
| Good | 44 (32.1%) |  |
| Fair | 0 (0.0%) |  |
| Poor | 0 (0.0%) |  |
| **Willing to attend the workshop in the future** | | |
| Yes | 132 (96.4%) |  |
| No | 4 (2.9%) |  |
| No reply | 1 (0.7%) |  |
| **Likelihood of recommending this workshop to a colleague or friend** | | |
| Very likely | 106 (77.4%) |  |
| Likely | 27 (19.4%) |  |
| Neutral | 3 (2.2%) |  |
| Unlikely | 0 (0.0%) |  |
| No reply | 1 (0.7%) |  |
| **The approach to finding out about this training workshop**^a^ | | |
| Organization's website | 26 |  |
| Email announcement | 42 |  |
| Social media | 49 |  |
| Referral | 41 |  |
| Others | 14 |  |

1. The question allow participants to make multiple-choice.
