# Supplementary material for: Global learning opportunities within social innovation in health (GLOWS): A modified Delphi process to identify and pilot core competencies for learning
Source: PLoS One. 2026 Jan 9;21(1):e0339359. doi: 10.1371/journal.pone.0339359 (PMC12788671; doi:10.1371/journal.pone.0339359)
Supplement: S7 Table — (DOCX) [file pone.0339359.s007.docx]

**S8 Table**: Full Consensus Statements

| **Preamble** |
| --- |
| 1.Social innovation can be defined as a community-engaged process that impacts health and social outcomes (Halpaap et al, Lancet GlobalHealth, 2020). Social innovation education and training can give local communities and health practitioners the key tools to solve problems relating to health. Currently limited evidence and consensus exists regarding core competencies that should be consistently achieved from social innovation in health training.  Although numerous education programmes exist globally, content and methods of delivery vary significantly, and most have been developed in the context of high-income countries.  The purpose of this survey is to identify social innovation learning competencies that can inform the delivery of teaching materials in social innovation in diverse settings, particularly resource constrained settings. The consensus statement which will be constructed from this project using modified Delphi methodology can be used by individuals learning about social innovation in health as well as research institutions, universities, and educators to build capacity for social innovation in health.  The primary target audience in this project is the individual learning about social innovation. We acknowledge that some competencies/mindsets/skills can be taught in a structured manner while others may be learnt from experiential learning or self-directed learning. Statements which refer to competencies that may be taught in structured social innovation training will be denoted with an * |
| **Mindsets** |
| 2.     **Empathy** refers to the ability to deeply understand and share feeling that the target audience/user of any given innovation targets. While empathy may be difficult to teach in some circumstances, it is important for learners to have an awareness of its importance and focus on developing empathy so that the beneficiaries’ needs are understood and met. |
| 3. **Resilience** refers to the ability to withstand difficulties and challenges. It refers to an ability to deal with failure and ambiguous circumstances. Developing resilience should be an important focus area for learners to develop in the context of social innovation in health and practical tools and guidance can be used to build resilience. |
| 4. **Adaptability** can ensure that social innovations are dynamic and thus can be implemented in different landscapes. Adaptability to rapidly iterate and respond to local contexts and needs is an important mindset to develop as part of social innovation training. |
| **Skills** |
| **Communication** |
| 5. **Pitching** is the ability to communicate verbally to gain support. Innovators who have better abilities to communicate their project, their needs and call for action assertively, have greater chances of success in seeking support. Pitching skills are therefore an important competency to become a competent social innovator. * |
| 6. **Community engagement** is is the process of working collaboratively with groups of people whether they are connected by geographic proximity, special interest, or similar situations to address shared issues. By developing community partnership-building competencies, social innovation trainees will be better equipped to navigate the local context, align diverse interests, and mobilize the necessary resources and support to bring their ideas to life. Engaging communities is a key skill to develop in social innovation training programmes. * |
| 7. **Storytelling** is used to connect people to real life experiences. It has the power to connect people, convey real human experiences, and inspire others to action. Storytelling is an important skill which should be included in social innovation training content. * |
|  |
| **Knowledge** |
| **Intersectionality** |
| 8. Training programs should equip social innovators with the skills to **engage respectfully with different cultural norms, values, and experiences.** This includes developing cultural sensitivity, communication strategies, and the ability to facilitate cross-cultural collaboration. Lessons on navigating diverse cultural contexts are essential in social innovation training. * |
| 9. Understanding **health disparities** from an intersectional lens is crucial for social innovators to create innovations that can truly address the needs of the most vulnerable populations. Understanding health disparities related to age, ethnicity and other intersectional issues should be a core theme in social innovation training. * |
| 10. The **social determinants of health (SDH)** are the non-medical factors that influence health outcomes. They are the conditions in which people are born, grow, work, live, and age, and the wider set of forces and systems shaping the conditions of daily life. Equipping trainees with the skills to analyse and identify the specific social determinants at play in a given context will enable them to design more targeted, impactful solutions. This type of systems-level thinking, and root cause analysis is essential for social innovators to move beyond surface-level interventions and drive sustainable, systemic change. Skills to identify the social determinants that cause these health disparities are important to teach as part of social innovation training. * |
| 11**. Prioritizing indigenous talents** and wisdom sends a powerful message about the core values of social innovation which are community-centricity, co-creation, and a deep respect for local context and expertise. Training programs should emphasize the importance of humility, respect, and a willingness to learn from local community members. Social innovators must be equipped with the mindset and skills to identify, uplift, and collaborate with indigenous leaders, knowledge holders, and change-makers. Prioritizing indigenous talents, strength, and wisdom of local communities should be a core message in social innovation training. * |
| 12.Learning how to effectively **engage with community members, conduct needs assessments**, and gather insights about the key issues and pain points experienced by the target population is essential. Identifying needs/challenges of the community so that social innovations can be tailored to meet these needs is an important skill which should be learnt. * |
| **Research and Evaluation Skills** |
| 13. Teaching **evaluation of social innovation/impact assessment** including how to develop metrics and indicators that key stakeholders can understand, and value should be a component of training. * |
| 30. **Research skills including conducting literature reviews** are an important competency in social innovation in health. These skills include search strategies, accessing and interpretation of literature. * |
| 14. **Manuscript writing,** and publication (writing for scientific publication) can help to demonstrate legitimacy of social innovations in order to gain support. Manuscript writing is a key skill to learn to be a competent social innovator. * |
| 15. Effective media use (including social media) for information dissemination and includes skills like content creation, social media management, digital marketing, and data analytics. It should be an important learning objective in social innovation. |
| **Entrepreneurship** |
| 16. **Navigating funding pathways** such as grant writing, securing investment and business model knowledge is an important skill for social innovators to develop. * |
| 17. Lessons on **building and managing a team** may include content on topics like team formation, communication, conflict resolution, and performance management can equip social innovators with the competencies to build and lead high-functioning, interdisciplinary teams and should be part of social innovation in health learning content. * |
| 18. Sustainable innovations are solutions that can be maintained, replicated, and scaled to drive significant, durable change. Teaching on **creating sustainable innovation** that will be long-lasting is an important learning competency. * |
| 19. Lessons on **network management can** cover topics like maintaining effective communication, coordinating joint activities, and sustaining long-term collaborations. How to build and maintain networks/relationships is something that should be included in social innovation learning content. |
| **Participatory Methods** |
| 20. **Crowdsourcing** is an approach in which a group of individuals attempt to solve all or part of a problem, then implement exceptional solutions in the community. It leverages the collective knowledge, creativity, and problem-solving abilities of a diverse group of individuals to tackle complex challenges. Crowdsourcing to identify social innovations is a useful tool to teach. * |
| 27**. Community based participatory research is** reliant on building community partnerships and co-creation of solutions. Co-creation of solutions with the people who will be impacted can build trust, ownership, and buy-in from the target community. It can leverage existing community assets, resources and ensure accessibility, relevance, and long-term sustainability of innovations. By making community engagement a core part of the training process, social innovators can cultivate essential skills like stakeholder mapping, facilitation, conflict resolution, and collaborative problem-solving. Community based participatory research is an important approach to achieving social innovation in health competencies. * |
| **Ethics and Advocacy** |
| 21. Learning materials should provide in-depth coverage of relevant **ethical frameworks, guidelines, and best practices** to ensure social innovations in health are designed and implemented responsibly. This knowledge helps social innovators identify potential ethical risks, make well-reasoned decisions, and establish clear accountability measures. Integrating ethical considerations also fosters a deeper sense of social responsibility and commitment to the wellbeing of the communities served. Ethical considerations and pathways should be included in social innovation in health training. |
| 22.  **Advocacy tools may** include stakeholder mapping, policy analysis, lobbying and campaigning, coalition building, and communication/messaging techniques. By cultivating advocacy competencies, social innovation training programs can help learners become more effective at influencing decision-makers, shifting mindsets, and mobilizing support for their health-focused initiatives. Advocacy tools should be taught to overcome barriers in local health systems to optimize success of social innovations. * |
| 29. Learning how to **navigate government policy including regulatory landscapes** is an important skill to be included in social innovation in health training. |
| **Practical Tools** |
| 23. **User centred design** is the iterative design process in which designers focus on the users and their needs in each phase of the design process. Teaching user centred design is important to deliver comprehensive social innovation training* |
| 24. Although **mentoring** one-to-one can be difficult practically especially if resources are limited, it allows for tailored feedback, troubleshooting, and the cultivation of essential soft skills that are difficult to impart through classroom-based instruction alone. One-to-one or alternatively group mentoring is important to achieve core learning competencies. |
| 25.   **Prototyping** allows social innovators to quickly test and iterate on their ideas, gather feedback, and refine their solutions before investing significant resources. Prototyping is a key skill to learn in social innovation training. * |
| 26. Using **generative play** i.e. playful activities and games to stimulate creativity and curiosity can be a useful tool to achieve important learning competencies in social innovation in heal |
| 28. **Definitions, theories, frameworks, case studies** are all key tools to achieve key social innovation competencies and skills. It is important to have resources that clarify what sets social innovation apart from other innovations by providing theoretical underpinnings. * |
