# Supplementary material for: Global learning opportunities within social innovation in health (GLOWS): A modified Delphi process to identify and pilot core competencies for learning
Source: PLoS One. 2026 Jan 9;21(1):e0339359. doi: 10.1371/journal.pone.0339359 (PMC12788671; doi:10.1371/journal.pone.0339359)
Supplement: S8 File — (PDF) [file pone.0339359.s008.pdf]

# Core competencies for social innovation in health training: Evidence from a global scoping review and crowdsourcing open call

Zixuan Zhu, Ogechukwu Benedicta Aribodor, Jamie Lynn Conklin, Yusha Tao, Emily Wallace, Iheukwumere Chidiogo Marigold,Kovey Mawuli,Amel Belkacemi, Jana Deborah Mier, Wenjie Shan, Gifty Marley, Anita walker, Komang Gde Ardi Pradnya Septiawan, Andrew Hamuza, Abigail Mier, Halpaap, Beatrice,Angelica Rocio Borbon Orjuela, Joel Francis,Adriana Patricia Saboya Ruiz,Gloria Aidoo-Frimpong,Jackie Nono, Marlita Putri Ekasari, Obidimma Ezezika, Rayner Tan, Bertha Chakhame, Per kaks, Jackeline Alger, Linet Mutisya, Nwadiuto O. Azugo, Allan Ulitin, Ángela Karina Sarria Caicedo, Fatema Ahmed, Tina Fourie, Elizabeth Chen, Joseph D Tucker

# Abstract

**Introduction**

Social innovation in health is a community-engaged process to stimulate creative solutions to local health challenges.  Although it has expanded , little is known about core competencies required for training social innovation in health. This evidence synthesis included data from a global scoping review and crowdsourcing open call to identify core competencies in social innovation in health training.

**Methods**

We organized a scoping review and a crowdsourcing open call to identify existing studies on training practices in social innovation in health. The scoping review followed PRISMA guidelines and the protocol was uploaded on the Open Science Framework (OSF). We extracted studies from 2007 and 2023 describing training on social innovation in health from nine databases. The purpose was to solicit training approaches for social innovation in health, focusing on  low and middle-income country (LMIC) settings . Textual data describing social innovation learning competencies from the open call and studies from the scoping review were descriptively analysed.

**Results**

Six skills, six mindsets and four types of knowledge were categorized as core competencies from 20 scoping review studies and 38 eligible open call entries. Seven competencies were further addressed for their frequent presence in the social innovation in health training. Communication skill(12 studies and 18 entries) and collaborative mindset(15 studies and 19 entries) were

highlighted in training to connect stakeholders and partners from local community; Community-engaged participatory methods(14 studies and 16 entries), and intersectional knowledge (9 studies and 13 entries)were considered crucial in understanding the health context and designing the solutions.

**Interpretation**

Our evidence synthesis identified a list of core competencies in social innovation in health using a scoping review and crowdsourcing open call. Future training programs or health education curricula designs could consider integrating the core competencies in developing standardized learning frameworks of social innovation in health.

58

# Introduction

Social innovation refers to new ideas that meets the social needs(1). Social innovation in health is a community-engaged process to address local health and social problems. (2) Social innovations provide a ground-up approach to developing new health services, engaging communities, and spurring social change. For example, a community-led telephone referral line in Malawi scaled up to provide nationwide maternal health services,(3) while a pay-it-forward sexually transmitted infection (STI) testing program in China leveraged monetary and non-monetary resources to support men who have sex with men (MSM). (4) The expanding social innovation in health movement underlines the need for training in this field.

Training and capacity strengthening are critical for advancing social innovation in health. However, there is limited focus on the content or format of core competencies, defined as skills, mindsets, and knowledge required to engage in and promote social innovation in health effectively. Many social innovation training programs are designed and promoted by researchers from high-income countries, with less focus on low- and middle-income countries (LMICs) settings. (5,6) Recognizing these gaps, the Special Program for Research and Training in Tropical Diseases (TDR) and the Social Innovation in Health Initiative (SIHI) have emphasized the need to identify training materials for social innovation in health.

This evidence synthesis aimed to identify and analyze the core competencies essential for social innovation in health using a scoping review and a global crowdsourcing open call. A crowdsourcing open call is a collective process engaging both experts and non-experts to solve

problems and share solutions(7). The data can then be used to inform courses, learning modules, and training workshops in this growing field. .

## Methods

The study was organized by the Social Innovation in Health Initiative (SIHI) in partnership with Social Entrepreneurship to Spur Health (SESH), the United Nations Children's Fund (UNICEF), the United Nations Development Programme (UNDP), the World Bank and the World Health Organization Special Programme for Research and Training in Tropical Diseases (TDR). SESH is an initiative to promote more creative, equitable, and effective health services using crowdsourcing open calls and other social entrepreneurship tools. We used a scoping review followed by a global crowdsourcing open call. The scoping review examined social innovation in health training and educational activities, synthesized existing evidence to provide an overview on the field. The scoping review findings informed the structure of the open call. The crowdsourcing open call invited submissions from the public and community—particularly from LMICs and social innovation networks—focusing on learning modules, teaching approaches, and content related to teaching social innovation in health to captured diverse perspectives. The protocol for the scoping review was co-developed by two early career researchers (OG and JC) and two senior researchers (EC and JT).

The scoping review revealed limitations in the amount and geographic scope of existing studies, with most research conducted in higher education settings in Europe and American regions. To

address this bias and enhance inclusivity, the open call was structured to specifically capture social innovation in health training in LMICs.

**Scoping Review**

This scoping review followed the Preferred Reporting Items for Systematic Reviews and Meta-Analyses (PRISMA) extension and the Joanna Briggs Institute (JBI) methodology for scoping reviews.(8,9) The JBI methodology strengthens the study's reliability by ensuring a systematic and transparent approach to literature selection and data extraction, minimizing bias and enhancing the comprehensiveness of the review.(9) The protocol was uploaded on the Open Science Framework (OSF), an open-access platform.(10)

**Eligibility Criteria**

Studies were included if they met any of the following criteria: 1) discussed how individuals learn or teach social innovation in health; 2) discussed methods for learning social innovation in health; 3) discussed social innovation in health within educational settings, such as universities (public or private); or 4) presented frameworks or methodologies related to social innovation in health. All study designs, publication types, and publication dates were included. Studies not published in English were excluded because of empirical data suggesting that this approach does not impact outcomes.(11,12) Relevance was determined based on explicit mentions of training, teaching, or learning competencies in the context of social innovation in health.

**Information Sources and Search Strategy**

A health sciences librarian (JC) searched the following nine databases on August 14, 2023: PubMed, APA PsycInfo (EBSCOhost), CINAHL Plus with Full Text (EBSCOhost), Cochrane Library (Wiley), Education Database (ProQuest), Education Full Text (EBSCOhost), Embase (Elsevier), ERIC (EBSCOhost), and Scopus (Elsevier). In addition, the research team searched the European Social Innovation Database, the SIHI website, and Google Scholar for relevant references. PubMed and Embase were chosen for their comprehensive health-related coverage, while ERIC and Education Full Text were included to capture educational resources relevant to the focus on competencies. We checked the citations of the included references and consulted with domain experts.

The search strategy incorporated both subject headings and keywords related to three main concepts: social innovation, education, and health. When searching health-related databases, the keyword 'health' was excluded, and similarly, the term 'education' was omitted when searching education-focused databases. An English language filter was applied to the search. The full search strategy across all databases is in the supplementary materials (Appendix I). We attached a separate evaluation section for the scoping review studies (supplementary appendix III)

**Study Selection**

All references were imported into EndNote (version 21) and deduplicated. Unique records were then imported into Covidence systematic review software (Veritas Health Innovation, Melbourne, Australia, available at www.covidence.org) for screening and data charting. Two independent researchers screened titles and abstracts for eligibility, and conflicts were resolved by discussion with a third reviewer (EC, JT, JC). Subsequently, two researchers independently

screened full-text and conflicts through discussion or arbitration with a third researcher (EC, OA or JT).

## Data Analysis

After reviewing all available studies, concepts were inductively categorized into three main domains—skills, mindsets, and knowledge—based on recurring themes identified across the studies. These categories were developed through a systematic process of grouping similar competencies and refining the classification to align with the core components of social innovation in health. After identifying a set of competencies, each included study was coded for the presence of these competencies, and whether they were present or absent was recorded. We also evaluated the evidence of scoping review for competencies by categories (see supplementary materials).

Data extraction was performed using Covidence, which was piloted with 100 references by two researchers (OG and EC). The standardized extraction form captured the following data: study characteristics (author, year of publication, title, country, setting, study purpose, teachers, learners, article type, duration of training, and framework or model), social innovation in health competencies (specific mindsets, knowledge, or skills), and teaching and training strategies (type, outcomes, and findings). Two reviewers (either OG, EC, ZZ, BW or KS) independently extracted data for each included reference, and a third reviewer assessed discrepancies (JT).

## Crowdsourcing Open Call

In addition to the scoping review, an open crowdsourcing call was organized on competencies and training practices in social innovation in health.  The open call was organized from February to April 2024.The open call was based on the TDR crowdsourcing open call practical guide (7) and was supported by the Social Entrepreneurship to Spur Health (SESH), TDR Global, and SIHI.

The open call organized a multisectoral steering committee comprising nine members from diverse geographic regions, seven of whom were from LMICs. Committee members were selected based on their expertise and experience in social innovation training, their residence in LMICs, and their prior involvement in social innovation in health training events. Five were training directors or had assisted in social innovation in health training events. Steering committee meetings were held biweekly via one-hour teleconference to update preparations, refine submission guidelines,  and provide expert feedback on the call for submission and promotion strategies.

Promotion of the open call was carried out through the SESH website, social media channels, email listservs, and partner organizations, especially through SIHI networks. These promotional channels were effective in reaching a wide audience. To ensure diversity in submissions, direct outreach was also conducted to key stakeholders in LMICs, including local organizations, training directors, and community leaders. Participants who had attended or organized social innovation in health training were encouraged to submit ideas related to the following: curriculum or module design for social innovation in health courses; adapting training practices in complex settings with limited teaching capacity, especially in LMICs; participatory training

methods (e.g., co-creation, flipped courses); and the evaluation of social innovation in health training. Submissions were accepted in any of the six UN languages: French, Spanish, Arabic, Russian, English, and Chinese.

Eleven independent judges,  including three steering committee members and eight external experts, were assigned to rate each submission on a 1-10 scale (with 1 being the lowest and 10 being the highest) based on five criteria. These included the following: clarity and of the idea; relevance to core learning competencies in social innovation in health; novelty of the training approach; feasibility, scalability, sustainability of the approach; and promotion of equity and fairness. Judges with conflicts of interest were recused. Non-English entries were initially examined for eligibility using translation tools before being judged. The final score was determined by averaging the five subcomponent scores, with a minimum score of 7 required for the selection of the finalists. Eight finalists were chosen, ensuing geographic diversity among the selected entries.

**Data Analysis**

For open call submissions, a descriptive analysis approach was used to identify recurring competencies, patterns, and relationships across the selected submissions. Findings were summarized in alignment with the research questions and objectives. Competencies with the highest frequency (over 20) in each domain (skills, mindsets, knowledge) were presented in the manuscript.

The scoping review studies, together with the open call entries, were synthesized by the research team into draft competencies, marking the transition to the next stage of the competency development project.

# Results

**Scoping review**

There were 20 studies included in our scoping review (Figure 1). Ten studies were from high-income countries, seven from middle-income and three from low-income countries. Two were published before 2010, nine were published between 2010 and 2019, and nine were published after 2020. Most training occurred in high-income countries (n=13). The most common region for training was North America (n=11).  Ten studies were case studies. Four studies were theories, frameworks, or models.

Most studies focused on activities conducted in college or university settings (n=14) compared to healthcare settings (n=3), virtual settings (n=2), or other settings. Teachers included college or university faculty (n=12) and researchers (n=8). Learners were undergraduate students (n=7), graduate students (n=5), researchers (n=5), healthcare providers (n=3), and community members (n=3).

The training activities lasted years (n = 6), months (n=7), or weeks (n=3). The majority of the studies (n=16) applied a specific approach, framework, or model to their training activities. With regards to training strategies, the studies often reported team-based learning (n=13), participatory elements (n=12), lectures (n=7), designathons (n=3), and flipped classrooms (n=2). All studies

referenced teaching social innovation in health skills and mindsets, and most (n=17/20, 85%) of the studies described teaching social innovation health knowledge.

**Crowdsourcing Open Call**

The crowdsourcing open call received 43 submissions, of which 38 were eligible. Ineligible were not relevant (n=1), over length(n=2),video or graph(n=2).The entries were from 24 men and 14 women, mostly aged 28-47 (71%, n=27) and had a master's degree or above (73%, n=28). Among the 38 eligible entries, 34 (89%) were from LMICs, and four were from HICs. Most entries were from regions of the Americas, African, and West Pacific regions, with the top countries being the Philippines (n=7), Colombia (n=5), and Argentina (n=3). More details are provided in Table 1. Thirteen entries were submitted in Spanish, and 25 were submitted in English. Regarding the submission content, eight entries (22%) were descriptions of social innovation in health training curriculums, and seven (19%) focused on the theories, frameworks, or models of social innovation in health training strategies. Thirty-six out of the 38 eligible open call entries were further processed into the data extraction stage to identify the key competencies related to social innovation in health competencies. Twelve entries received an average score of 7/10 or higher (Figure 2).

**Social innovation in health competencies across scoping review and open call**

We identified 16 core competencies related to social innovation in health by pooling the evidence from both scoping review and open call. Competencies were categorized into three domains: skills (six competencies), mindsets (six competencies), and knowledge (four competencies) (Figure 3). Seven competencies were further discussed in the following sections

as the most frequently presented and commonly acknowledged competencies across the evidence of each domain, including problem-solving skills, communication skills, creative thinking, collaboration mindsets, community-engaged participatory method, health disparity knowledge, and intersectionality on health, gender, and local culture. We conducted subgroup analysis by isolating evidence from only high-income settings and low- and middle-income settings. The subgroup results are similar regarding the frequencies of evidence of seven core competencies (supplementary). The other competencies were categorized and listed in Table 2.

**Social innovation in health skills**

Problem-solving skills were the most common frequently identified core competency. We define problem-solving as the ability to frame local health challenges and tackle them. Strong problem-solving skills were mentioned in 20 studies and 28 of the open-call entries (13–32). One study demonstrated how creative problem-solving combined with human-centred design enabled participants to develop user-oriented health solutions, resulting in improved access to healthcare services (18). Another study emphasized problem-solving through entrepreneurial teamwork (25). Problem-solving skills were particularly important in resource-limited settings such as rural areas. Six entries were curricula that included problem-solving skills. Effective problem-solving could foster resilience in dynamic and resource-limited environments, enabling innovators to adapt to emerging challenges while focusing on sustainable impact.

Communication skills also emerged as a critical competency in social innovation. Defined as the ability to effectively connect with a broad range of stakeholders, especially people with lived experience and those who may be potential partners (18), communication skills were empathized

in 12 studies (13,15,18,19,22–24,26,28–30,32) and 18 open call entries. Design thinking and participatory processes with stakeholders were applied in LMIC settings to ensure culturally sensitive interventions(28). Involving community members in co-creating health solutions contributed to the trust-building process.  Interventions were more widely accepted, increasing engagement and participation in health programs. One study used human-centered design strategies to enhance communication between researchers and local populations (18). Communication skills allowed researchers and innovators to address needs and concerns during the solution design process fully. These have also been applied to the prototype development training to ensure the pathway for iterative feedback of solutions in open call cases. The ability to communicate effectively engaged stakeholders, fostered collaboration, facilitated knowledge transfer, and further ensured that interventions were clearly understood

**Social innovation in health mindsets**

The most common mindset-related learning competency identified was the need for creativity and novelty in developing health solutions centred and implementation strategies. Creativity is defined as the ability to think divergently and develop innovative approaches when traditional methods are insufficient. This mindset was highlighted in 16 studies and 22 open call entries (13–15,18–21,23,25–32). One study introduced an entrepreneurial pitch project where students were tasked with designing technological and social innovations for health challenges in LMICs(20). This experiential learning approach significantly enhanced students' ability to apply creative thinking in real-world global health contexts, driving them to creatively address resource shortages in underserved areas by leveraging community assets. Creativity formulated an open mind for creative solutions when traditional approaches fell short of addressing the healthcare

challenge in resource-limited settings. In open call entries, 13 case studies have demonstrated innovative solutions and how they were generated and implemented to address health problems. Embracing innovative thinking could drive the development of groundbreaking solutions to address complex health challenges, promote the adaptation of new approaches to meet evolving needs, and ensure that interventions remain relevant and effective in a rapidly changing healthcare landscape.

Another important learning competency was collaborative mindsets, which we defined as the ability to partner with stakeholders, leverage network resources, and engage in interdisciplinary teamwork. Fifteen scoping review studies and 19 open call entries highlighted the importance of collaboration, especially with interdisciplinary teams to address complex health challenges (13,14,16,18–20,22–26,29–32). Effective collaboration with stakeholders and the community integrated diverse expertise and perspectives, which was essential for tackling multifaceted health issues. For instance, one study illustrated how interdisciplinary collaboration among epidemiologists, community health workers, and local leaders significantly enhanced rural health initiatives targeting vector-borne diseases (30). Another study demonstrated that cooperation between engineers, public health experts, and community stakeholders led to more effective and sustainable water quality management interventions (29). One open call finalist entry of public servants in health departments also emphasized their role as natural leaders across the various sectors that influence health and promote social innovation through a multidisciplinary and integrated approach, fostering active participation and effective inclusion. This approach improves problem-solving capabilities and ensures that health solutions are more comprehensive and adaptable, addressing immediate and long-term needs.

**Social innovation in health knowledge**

Community-engaged participatory methods emerged as a critical knowledge competency in social innovation learning. We define community-engaged participatory methods as the methods to engage community stakeholders as full partners in all phases of research(33). These methods include community advisory boards, qualitative research, co-creation, open calls, and design-a-thons. Fourteen scoping review studies and 16 open call entries discussed the need to learn community engagement strategies, especially the ones relevant to resource-poor settings, such as community advisory boards, qualitative research, co-creation, open calls, and design-o-thons (7,13,14,16–18,20–23,26,28–31). One study demonstrated the effectiveness of participatory workshops in addressing gender-related health issues, where co-created solutions led to greater acceptance and long-term adoption (16). Other cases included using cross-disciplinary collaboration to involve community members in planning health initiatives through focus groups and meetings(17). This co-creation strategy had built trust and improved participation, aligning interventions with the cultural and social needs of the communities and leading to better health outcomes. Open call entries have further provided cases of implementation of community engagement strategies where the strategies were developed with local culture, labour, and resources to maximize the acceptance of the strategy among community members and maximize the mobilization of resources. One of the open-call finalist entries integrated the co-creation strategies with the historical and cultural elements of the local Filipino community (34). These strategies fostered collaboration and ensured that interventions were tailored to the specific needs of the communities they serve.

Understanding health disparities was another essential knowledge competency. For social innovation in health. Nine scoping review studies and 14 open call entries discussed knowledge of health disparities, often at the systems level (16,18,20,22–24,29–31). One study emphasized how medical education can address health disparities by incorporating experiential learning to help students engage directly with marginalized communities (24). This approach enabled future healthcare professionals to understand the social determinants of health better and develop more culturally sensitive interventions. The result was an increased capacity to address inequities through tailored interventions that resonate with the specific needs of underserved populations. Open call entries also underlined the awareness of health disparities and exploration of their root as a fundamental element to targeting innovative solutions towards specific groups, which further merged in training practices in social innovation in health. One entry highlighted the inequality of health access for families living in sanitary vulnerability without access to main water and brought it into the co-creation process for technology development. Recognizing these disparities was crucial for developing equitable health interventions that address the underlying social, economic, and structural factors contributing to unequal health outcomes across different populations.

Understanding intersectionality was identified as an important core competency of social innovation. Intersectionality is a concept that describes how a person's identity can lead to different experiences of discrimination and privilege(35). The intersectional lens provided social innovators with an understanding of culture or gender-specific needs and approaches for creating health solutions that were relevant and effective in different cultural settings, and for addressing issues related to gender and health equity. Nine of the scoping review studies and 12 open call

entries called for an understanding of local culture to create health solutions that are both relevant and effective(16,18,22–25,29–31). For example, one study used participatory workshops to challenge gender stereotypes and co-design culturally appropriate health programs with community members, particularly women(16). By engaging community members, particularly women, in co-designing health interventions, the study challenged harmful norms and ensured that health programs were culturally appropriate and inclusive. This approach fostered a sense of ownership and trust, leading to higher levels of participation from marginalized groups. The same emphasis on the intersectionality of gender has also been repeated in the open call entries, as five case studies in the social innovation in health training have been designed for women or illustrate the topics of gender perspective, sexual and reproductive health, and maternal care. These studies also highlighted the importance of addressing gender stereotypes within cultural contexts to promote equitable health outcomes and avoid reinforcing harmful norms that may limit access to healthcare for women and marginalized groups.

## Discussion

This study is identified the core competencies of social innovation in health training with evidence from a scoping review and a crowdsourcing open call.  Our findings revealed key insights and gaps in the literature on social innovation in health core competencies for learning. Sixteen core competencies for learning were categorized in social innovation in health skills, mindset, and knowledge, especially focusing on problem-solving skills, communication skills, creative thinking, collaboration mindsets, community-engaged participatory methods, etc. This study extends the literature on social innovation in health training by combining data from a

scoping review and a global open call, using participatory crowdsourcing approaches to identify LMIC evidence, and focusing on learning and teaching related to social innovation in health.

Our data demonstrated that community engagement elements are important for social innovation in health training. Multiple competencies identified in our study focused on community engagement in training: community representatives as key partners of collaboration, community members as learners of training, and community-engaged participatory methods applied for training. These findings align with previous toolkits on crowdsourcing participatory method (7) and WHO guidelines on community engagement (36). Community engagement allows targeted and accessible health training to promote positive health impact and training outcomes. Future social innovation training programs should deepen community engagement to realize the full potential of local knowledge and resources.

Our data highlighted the importance of incorporating intersectionality into social innovation training. Intersectionality, which examines how overlapping identities influence experiences of discrimination and privilege, is increasingly recognized in the broader health education literature (37,38). Understanding intersectionality equips learners to address healthcare inequities by tailoring solutions to the unique needs of diverse populations. For instance, an intersectional lens can help identify and dismantle systemic barriers disproportionately affecting marginalized groups. From an equity perspective, this competency is essential for promoting fair and inclusive healthcare interventions.

Our subgroup analysis revealed the similarity of training focus across high-income settings and low and middle income settings, which provided a grounded basis for the further generalization and promotion of these core competencies globally. The team applied a Delphi method to build consensus based on the evidence synthesis finding. The consensus aims to develop agreements on the core competency across borders. Our study also informed the design of social innovation in health midyear training workshops[1]. The open-call finalists, social innovation in health experts who joined the workshop co-creation, and trainees who attended the workshops were also invited into the consensus-building process. The consensus concluded with a core competencies list of nine items from three domains.

**Limitations**

While the open call opportunity was disseminated through various channels, there may have been selection bias due to snowballing dissemination within our research networks and institutional collaborations (e.g., universities, the Social Innovation in Health Initiative). Although the Social Innovation in Health Initiative (SIHI) is a well-established global network, the reliance on these connections may have inadvertently limited the diversity of entries. To mitigate this, we allowed submissions in all six UN languages. To address this limitation, the multilingual open-call entries provided some representation from these regions. Despite the limitations, this study provides valuable insights into core competencies for learning for social innovation in health and highlights areas for further research and development in LMIC contexts

[1] https://www.seshglobal.org/sihi-training-workshop-2024/

**Implications**

Our study has implications for learning, practice, and research. From a learning perspective, the core competencies could be used to complement existing social innovation curricula (e.g., Uppsala University) or to develop new social innovation courses. Additional training syllabi need to be developed and aligned with these competencies, integrate training design with real-world training focus. In practical application, the Social Innovation in Health Initiative (SIHI) used these competencies to structure its Social Innovation in Health Mid-Year Training Workshop in the summer of 2024. The eight final open call groups were further invited into the co-creation process of SIHI midyear training workshops to implement the training ideas and, optionally, to engage in the modified Delphi-based consensus-building process. From a research perspective, since most of the existing studies evaluating social innovation have been short-term(6),there is a need to assess medium and long-term outcomes associated with learners who join social innovation in health courses.

## Conclusion

This study is an important first step in developing universal social innovation in health competencies for future health research and practice. The evidence identified through the open call and scoping review provided a descriptive overview of core competencies for learning in social innovation in health. Future research and training practices based on the competencies are needed to provide better guidance on social innovation in health training.

Figure 1. PRISMA diagram for social innovation in health competency scoping review

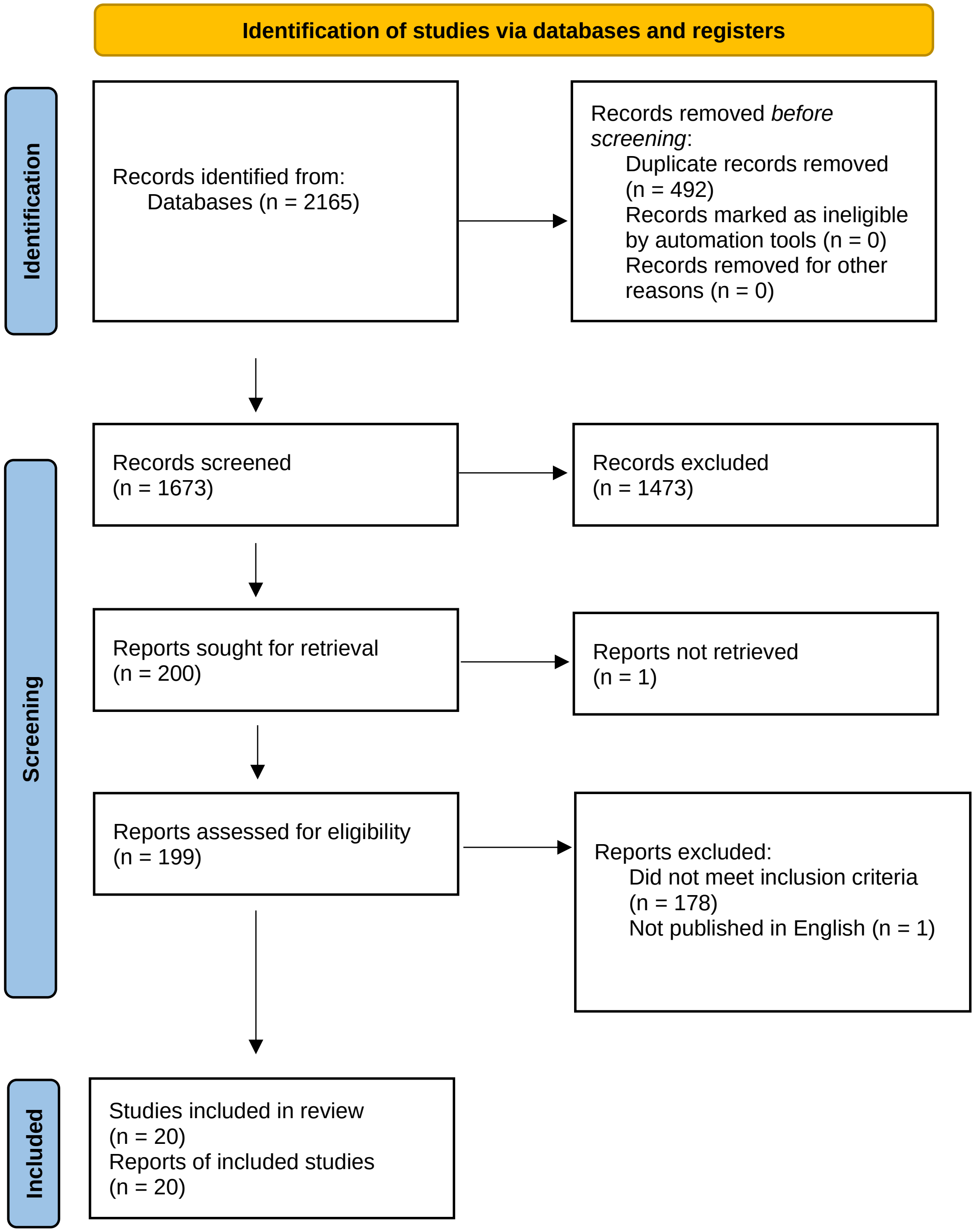

1

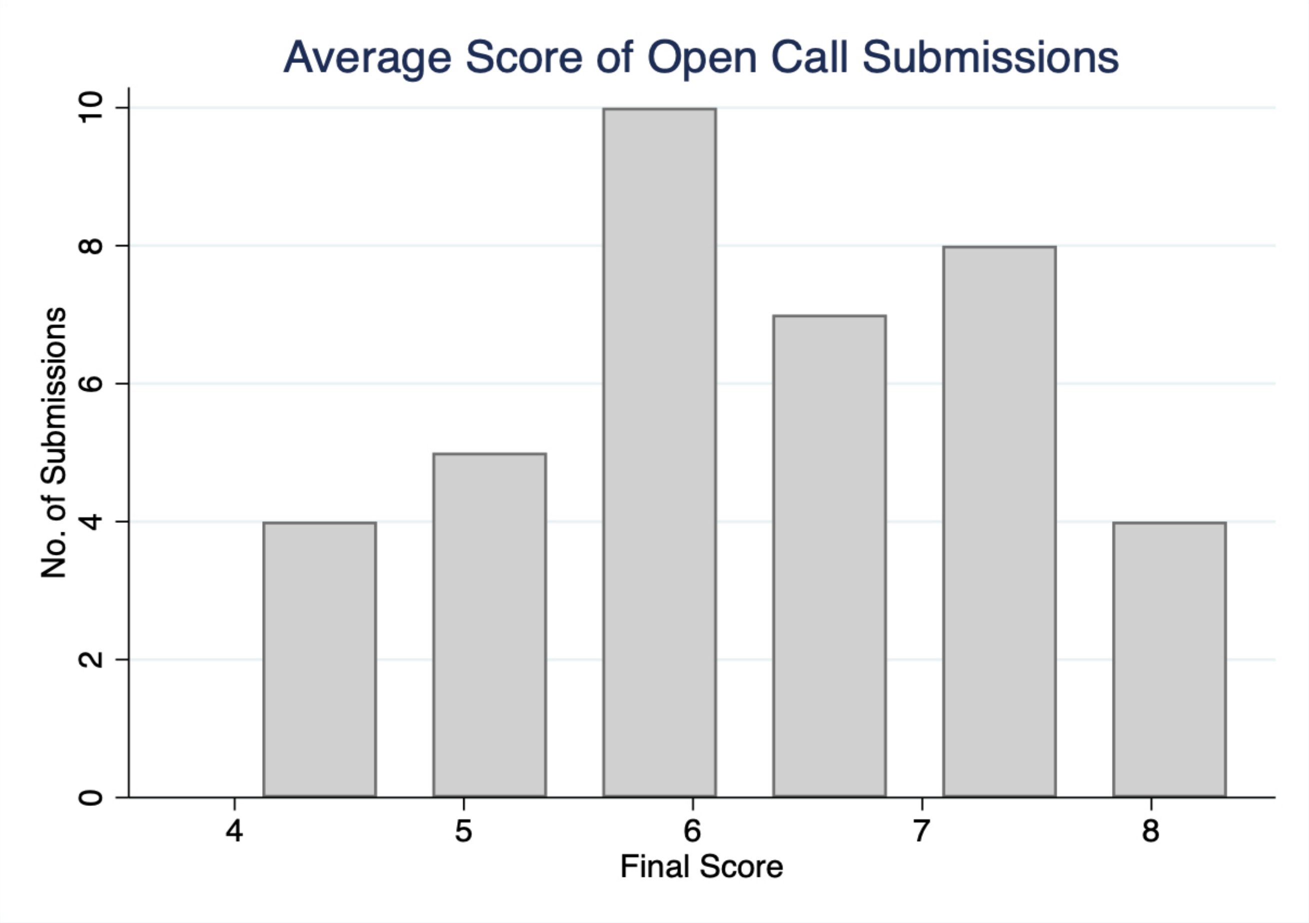


**Figure 2. Distributions of the Average Score of Open Call Entries.**

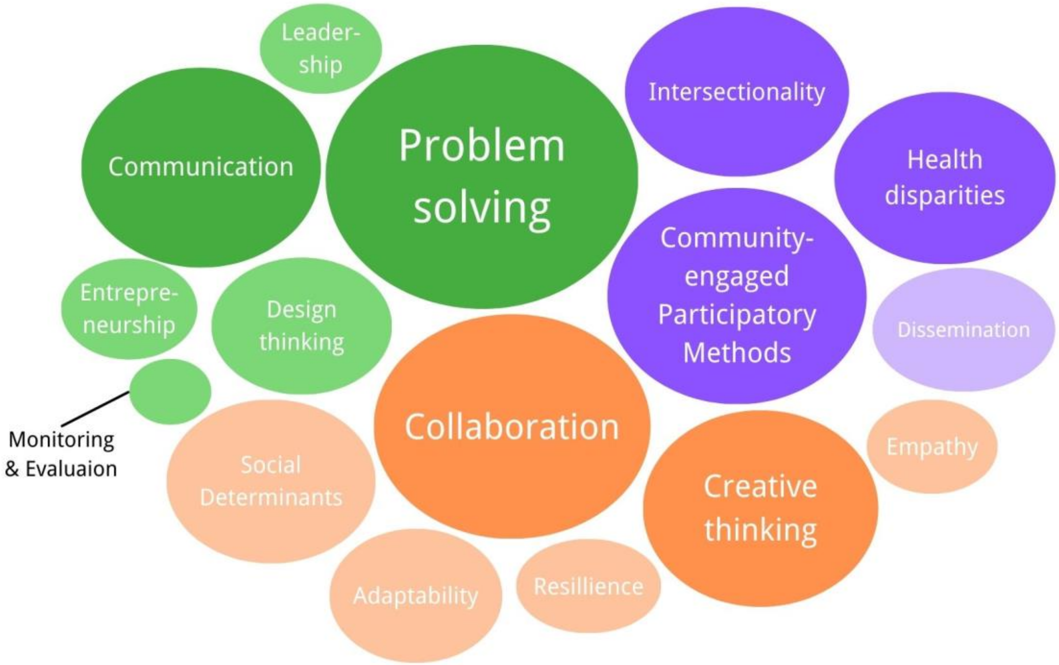


**Figure 3. Skills, mindset and knowledge identified from the evidence**

**Table 1: Socio-demographic characteristics of "Social innovation in health learning competencies" open call participants (N=38)**

| ***Age*** | n | % |
|---|---|---|
| 18-27 | 4 | 10.5% |
| 28-37 | 16 | 42.1% |
| 38-47 | 11 | 29.0% |
| 48 years old or older | 7 | 18.4% |
| | | |
| ***Gender*** | | |
| Male | 24 | 63.2% |
| Female | 14 | 36.8% |
| | | |
| ***Education level*** | | |
| No formal schooling completed | 1 | 2.6% |
| High school graduate, diploma or the equivalent (for example: GED) | 1 | 2.6% |
| Bachelor's degree | 8 | 21.1% |
| Masters or similar professional degree | 22 | 57.9% |
| Doctoral degree | 6 | 15.8% |
| | | |
| ***Income level by World Bank*** | | |
| Low income | 4 | 10.5% |
| Middle income | 30 | 79.0% |
| High income | 4 | 10.5% |
| | | |
| ***WHO Region*** | | |
| Region of the Americas | 17 | 44.7% |
| African Region | 10 | 26.4% |
| Western Pacific Region | 9 | 23.7% |
| European Region | 1 | 2.6% |
| South-East Asian Region | 1 | 2.6% |
| | | |
| ***Country of residence*** | | |
| Philippines | 7 | 18.6% |

| | | |
|---|---|---|
| Colombia | 6 | 15.9% |
| Argentina | 3 | 7.9% |
| Nigeria | 3 | 7.9% |
| Kenya | 2 | 5.3% |
| China | 2 | 5.3% |
| Uganda | 2 | 5.3% |
| Liberia | 1 | 2.6% |
| Canada | 1 | 2.6% |
| Brasil | 1 | 2.6% |
| Cameroon | 1 | 2.6% |
| Panamá | 1 | 2.6% |
| South Sudan | 1 | 2.6% |
| Bolivia | 1 | 2.6% |
| Paraguay | 1 | 2.6% |
| Ecuador | 1 | 2.6% |
| Honduras | 1 | 2.6% |
| USA | 1 | 2.6% |
| India | 1 | 2.6% |
| Ireland | 1 | 2.6% |
| **Total** | **38** | **100%** |

**Table 2. Social innovation in health competencies included across scoping review and open call**

| Type of competencies | Competencies | Findings from scoping review/open call | Number of articles from scoping review that mention competency (n) | Number of open call entries that mention competency (n) | Number of scoping review articles and open call entries that mention competency (n) |
|---|---|---|---|---|---|
| Skills | Problem solving | Focus on a problem-solving orientation to investigate and frame challenges that directly address local problems | 20 | 28 | 48 |
| Skills | Communication | Enhance communication skills and effectively communicate with a broad range of communities, especially people with lived experience and potential partners. | 12 | 18 | 30 |
| Skills | Design thinking | Understand the basics of design thinking principles to incorporate end-user feedback across the life of a social innovation | 7 | 20 | 27 |
| Skills | Entrepreneurship & Sustainable financing | Strengthen entrepreneurial skills to raise funds for social innovation, develop innovative financing and sustainability approaches, and rapidly iterate ideas. | 9 | 10 | 19 |
| Skills | Leadership | Build leadership skills to nurture relationships with the local community, enhance multi-stakeholder partnerships, and manage diverse groups. | 12 | 5 | 17 |

| | | | | | |
|---|---|---|---|---|---|
| Skills | Monitoring & Evaluation | Monitor and evaluate the effectiveness of social innovations in health | 4 | 13 | 17 |
| Mindset | Creative thinking | Generates creative and novel solutions | 16 | 22 | 38 |
| Mindset | Collaboration& Partnership | Collaborate with partners to conduct interdisciplinary teamwork to achieve comprehensive health solutions | 15 | 19 | 34 |
| Mindset | Social determinants | Recognize social determinants and contextual factors in health. | 11 | 18 | 29 |
| Mindset | Adaptability | Practice adaptability so they can rapidly iterate and respond to the local context and feedback | 10 | 18 | 28 |
| Mindset | Empathy | Build empathy to connect deeply with local communities of interest | 10 | 6 | 16 |
| Mindset | Resilience | Build a growth mindset to manage and expect failures, learn over time, and develop resiliency | 3 | 4 | 7 |
| Knowledge | Community Engaged Participatory Methods | Learn community engagement strategies that are relevant to resource-poor settings, including community advisory boards, qualitative research, co-creation, open calls, and designathons | 14 | 16 | 30 |
| Knowledge | Health care disparities | Understand health disparities and how they are rooted in intersectional inequities. | 9 | 14 | 23 |
| Knowledge | Interlens of culture context/gender | Understand culture specific/gender specific needs and approaches for creating health solutions that are relevant and effective in different cultural settings, and for addressing issues related to gender and health | 9 | 13 | 22 |

| | | | | | |
|---|---|---|---|---|---|
| | | equity | | | |
| Knowledge | Dissemination | Disseminate information about social innovations for a broad public audience through videos, infographics, publications, and related approaches | 5 | 10 | 15 |

# Reference:

1. Mulgan G, Tucker S, Ali R, Sanders B. Social Innovation: what it is, why it matters, how it can be accelerated. London: University of Oxford, Young Foundation; 2007.
2. Halpaap BM, Tucker JD, Mathanga D, Juban N, Awor P, Saravia NG, et al. Social innovation in global health: sparking location action. Lancet Glob Health. 2020 May 1;8(5):e633–4.
3. Blauvelt C, West M, Maxim L, Kasiya A, Dambula I, Kachila U, et al. Scaling up a health and nutrition hotline in Malawi: the benefits of multisectoral collaboration. BMJ. 2018 Dec 7;k4590.
4. Yang F, Zhang TP, Tang W, Ong JJ, Alexander M, Forastiere L, et al. Pay-it-forward gonorrhoea and chlamydia testing among men who have sex with men in China: a randomised controlled trial. Lancet Infect Dis. 2020 Aug 1;20(8):976–82.
5. Monteiro S, Isusi-Fagoaga R, Almeida L, García-Aracil A. Contribution of Higher Education Institutions to Social Innovation: Practices in Two Southern European Universities. Sustainability. 2021 Mar 24;13(7):3594.
6. Tao Y, Tan RKJ, Wohlfarth M, Ahumuza E, Aribodor OB, Cruz JRB, et al. Social innovation in health training to engage researchers in resource-limited settings: process description and evaluation. Health Promot Int. 2024 Apr 1;39(2):daae025.
7. World Health Organization, UNICEF/UNDP/World Bank/WHO Special Programme for Research and Training in Tropical Diseases. Crowdsourcing in health and health research: a practical guide [Internet]. Geneva: World Health Organization; 2018. Available from: https://iris.who.int/handle/10665/273039
8. Peters MDJ, Marnie C, Colquhoun H, Garritty CM, Hempel S, Horsley T, et al. Scoping reviews: reinforcing and advancing the methodology and application. Syst Rev. 2021 Dec;10(1):263.
9. Tricco AC, Lillie E, Zarin W, O'Brien KK, Colquhoun H, Levac D, et al. PRISMA Extension for Scoping Reviews (PRISMA-ScR): Checklist and Explanation. Ann Intern Med. 2018 Oct 2;169(7):467–73.
10. Conklin J, Yusha Tao, Li K, Liyuan Zhang, Linet M. Mutisya, Caicedo ÁKS, et al. Social Innovation in Health Learning Competencies: A Global Scoping Review of the Literature [Internet]. OSF Registries; 2023 [cited 2024 Nov 24]. Available from: https://osf.io/ynw8g/
11. Dobrescu A, Nussbaumer-Streit B, Klerings I, Wagner G, Persad E, Sommer I, et al. Restricting evidence syntheses of interventions to English-language publications is a viable methodological shortcut for most medical topics: a systematic review. J Clin Epidemiol. 2021 Sep;137:209–17.
12. Morrison A, Polisena J, Husereau D, Moulton K, Clark M, Fiander M, et al. THE EFFECT OF ENGLISH-LANGUAGE RESTRICTION ON SYSTEMATIC REVIEW-BASED META-ANALYSES: A SYSTEMATIC REVIEW OF EMPIRICAL STUDIES. Int J Technol Assess Health Care. 2012 Apr;28(2):138–44.
13. Backes DS, Colomé JS, Mello GBD, Gomes RCDC, Lomba MDLLDF, Ferreira CLDL. Social entrepreneurship in the professional training in Nursing. Rev Bras Enferm. 2022;75(3):e20220391.

14. Bennett AG, Cassim F, van der Merwe M. How design education can use generative play to innovate for social change: A case study on the design of South African children's health education toolkits. Int J Des. 2017;11(2):57–72.
15. Boore J, Porter S. Education for entrepreneurship in nursing. Nurse Educ Today. 2011 Feb;31(2):184–91.
16. Camussi E, Sassi C, Zulato E, Annovazzi C, Ginevra MC. Hacking women's health: A new methodology. J Prev Interv Community. 2020 Apr 2;48(2):174–88.
17. Chahine T. Toward an Understanding of Public Health Entrepreneurship and Intrapreneurship. Front Public Health. 2021 Apr 9;9:593553.
18. Chen Y, Roldan M. Digital innovation during covid-19: Transforming challenges to opportunities. Commun Assoc Inf Syst. 2021;48:15–25.
19. Cook R, Dawes D, Fanning A. Business and innovation first. Prim Health Care. 2012;22(2):20–1.
20. Ezezika O, Gong J. Experiential Learning in the Classroom: The Impact of Entrepreneurial Pitches for Global Health Pedagogy. Pedagogy Health Promot. 2021;7(2):118–26.
21. Gardner CA, Acharya T, Yach D. Technological and social innovation: a unifying new paradigm for global health. Health Aff Millwood. 2007;26(4):1052–61.
22. Gilmartin MJ. Principles and practices of social entrepreneurship for nursing. J Nurs Educ. 2013;52(11):641–4.
23. Heinze KL, Banaszak-Holl J, Babiak K. Social entrepreneurship in communities: Examining the collaborative processes of health conversion foundations. Nonprofit Manag Leadersh. 2016;26(3):313–30.
24. Khan N, Rogers A, Melville C, Shankar R, Gilliar W, Byrne P, et al. Using medical education as a tool to train doctors as social innovators. BMJ Innov. 2022;8(3):190–8.
25. Mehta K, Zappe S, Brannon ML, Zhao Y. REAL-WORLD SOLUTIONS. ASEE Prism. 2016;25(6):37.
26. Mier-Alpano JD, Cruz JRB, Fajardo MS, Barcena JF, Ekblad E, Hazell F, et al. Facilitating learning exchange and building a community of practice to accelerate social innovation in health. BMJ Innov. 2022;8(3):155–60.
27. Rai SD. Social entrepreneurship in nursing -- a tool for learning, advocacy and income generation. Singap Nurs J. 2007;34(3):7–13.
28. Skywark ER, Chen E, Jagannathan V. Using the Design Thinking Process to Co-create a New, Interdisciplinary Design Thinking Course to Train 21st Century Graduate Students. Front Public Health. 2021;9:777869.
29. Van Niekerk L, Echavarria MI, Alger J, Castro-Arroyave DM, Bautista-Gomez MM, Nieto Anderson CI, et al. Building the social innovation for health ecosystem in Latin America: Experiences and learning from SIHI-LAC. BMJ Innov. 2022;8(3):224–33.
30. Victor Meza V, Isabella Montero J, Catalina Mora F, Pablo Chiuminatto M, Arturo Grau D, Gabriela Carrasco R, et al. Social Innovation in Health. Int Conf High Educ Adv. 2022;2022-June:1109–16.
31. Wise S. Introducing MABL: A New Social Innovations Programme at the University of Melbourne. Child Aust. 2016;41(4):305–7.

32. Zidek L, Kauanui SK, Haytko DL. CROSS-LEVEL, CROSS-DISCIPLINARY, CROSS-CULTURAL COLLABORATIONS IN ACTION: A PUBLIC SERVICE EDUCATIONAL INITIATIVE INTEGRATING HEALTH, ENGINEERING AND ENTREPRENEURSHIP. Acad Educ Leadersh J. 2012;16(1):107–22.
33. Duea SR, Zimmerman EB, Vaughn LM, Dias S, Harris J. A Guide to Selecting Participatory Research Methods Based on Project and Partnership Goals. J Particip Res Methods [Internet]. 2022 May 23 [cited 2024 Nov 25];3(1). Available from: https://jprm.scholasticahq.com/article/32605-a-guide-to-selecting-participatory-research-methods-based-on-project-and-partnership-goals
34. Bersamira CS, Macaraeg J. Advocacy and Community Building to Address Filipino Behavioral Health. Hawaii J Health Soc Welf. 2022 Dec;81(12):338–40.
35. Crenshaw K. Mapping the Margins: Intersectionality, Identity Politics, and Violence against Women of Color. Stanford Law Rev. 1991 Jul;43(6):1241.
36. Community Engagement: A Health Promotion Guide for Universal Health Coverage in the Hands of the People. 1st ed. Geneva: World Health Organization; 2020. 1 p.
37. Routen A, Lekas HM, Harrison J, Khunti K. Intersectionality in health equity research. BMJ. 2023 Dec 29;383:2953.
38. Samra R, Hankivsky O. Adopting an intersectionality framework to address power and equity in medicine. Lancet Lond Engl. 2021 Mar 6;397(10277):857–9.

**Supplemental files list**

**Appendix I：Full search terms for PUBMED**

**Appendix I**:  PubMed Search Strategy

| Set # | |
|---|---|
| 1 | “social innovation”[tiab] OR “social innovations”[tiab] OR “social innovator”[tiab] OR “social innovators”[tiab] OR “social entrepreneurship”[tiab] OR “social entrepreneur”[tiab] OR “social entrepreneurs”[tiab] OR “social enterprise”[tiab] OR “social design”[tiab] OR changemaking[tiab] OR change-making[tiab] OR “change making”[tiab] OR changemaker[tiab] OR change-maker[tiab] OR “change maker”[tiab] OR changemakers[tiab] OR change-makers[tiab] OR “change makers”[tiab] |
| 2 | "Education"[Mesh] OR "education" [Subheading] OR "Learning"[Mesh] OR "Students"[Mesh] OR educat*[tiab] OR train*[tiab] OR teach*[tiab] OR learn*[tiab] OR curriculum[tiab] OR curricula[tiab] OR curricular[tiab] OR “staff development”[tiab] OR “professional development”[tiab] OR student[tiab] OR students[tiab] OR pedagogy[tiab] OR pedagogies[tiab] OR pedagogical[tiab] OR “academic performance”[tiab] OR “academic success”[tiab] OR competency[tiab] OR competencies[tiab] OR competent[tiab] OR workshop[tiab] OR workshops[tiab] OR class[tiab] OR classes[tiab] OR course[tiab] OR courses[tiab] |
| 3 | #1 AND #2 |
| 4 | #3 AND English[lang] |

## Appendix II: Evaluation outcomes across scoping review results

Across the 20 articles included in the scoping review, eight (40%) of them evaluated teaching/training outcomes associated with social innovation in health competencies.

We observed that only one study (5%) (Backes, 2022) evaluated all the key competencies—skills, knowledge, and mindsets. Two manuscripts (10%) (Gilmartin, 2013; Van Niekerk, 2022) assessed a combination of skills and knowledge.

### Evaluation of social innovation in health mindsets (attitudes)

Mindset is an established set of attitudes someone holds. Only 4 manuscripts (20%) evaluated social innovation in health mindsets, (Mehta, 2016; Heinze, 2016; Camussi 2020; Backes, 2022). The unifying outcome across these manuscripts is twofold: First, the recognition that fostering positive and inclusive mindsets, such as openness to change and resilience, is crucial for driving health innovation. These mindsets enable innovators and health professionals to engage effectively with diverse teams and stakeholders, thereby enhancing the implementation and sustainability of health solutions. Second, these manuscripts collectively underscore the importance of nurturing adaptability and a commitment to continuous learning. By cultivating these attitudes, individuals are better equipped to respond to the evolving challenges in healthcare and to contribute to the development of sustainable and innovative health practices.

The manuscripts provided insights into how various attitudes contribute to effective health innovation, each employing different methodologies: Mehta (2016), using mixed-methods that included surveys and qualitative interviews, revealed that students developed a proactive mindset characterized by enhanced global awareness and a strong inclination towards innovative problem-solving in health contexts. Similarly, Heinze (2016) employed case studies and longitudinal tracking of community health initiatives to show that fostering adaptability and trust within communities was crucial for effective collaboration and addressing local health issues. Camussi (2020), utilizing focus groups and behavioural assessments, demonstrated that participants developed inclusive attitudes, valuing diversity and cognitive engagement, which supported effective communication and collaboration across diverse groups. Notably, both Mehta (2016) and Camussi (2020) used qualitative elements to capture the depth of attitude changes. Finally, Backes (2022) employed surveys and participatory action research to reveal that self-empowerment and adaptability were key in developing a mindset geared towards continuous learning and proactive engagement in health innovation projects.

### Evaluation of social innovation in health skills (abilities)

We observed that only four manuscripts (20%) evaluated social innovation in health skills (abilities) (Gilmartin, 2013; Backes, 2022; VictorMeza 2022; VanNiekerk 2022). Across these manuscripts, we observed a unifying outcome: the recognition that equipping

individuals with a diverse and adaptable set of practical skills is crucial for effectively addressing the multifaceted challenges of health innovation. This skill development empowers professionals to work effectively in multidisciplinary teams, engage meaningfully with diverse stakeholders, and drive sustainable health solutions that can adapt to the ever-evolving challenges in healthcare.

These four manuscripts highlighted the importance of developing professional skills that are adaptable and applicable across various health domains, supporting the practical implementation of innovative health solutions. For instance, the role of multidisciplinary networks in fostering innovative thinking and professional skill development among young professionals and students was emphasized by Victor Meza (2022). The study specifically evaluated skills such as collaborative problem-solving, critical thinking, and ethical decision-making. Participants developed competencies in effectively working in diverse teams and navigating complex health challenges through innovative approaches. Van Niekerk (2022) demonstrated how relationships among stakeholders and participation in workshops and webinars enhanced practical knowledge and skills. The study focused on evaluating skills such as strategic planning, stakeholder engagement, and project management. These skills were found to be essential for sustaining healthcare innovations by ensuring that initiatives were well-organized, inclusive, and effectively implemented. Gilmartin (2013) assessed the development of practical skills through course assignments and found significant improvements in leadership, social entrepreneurship, and public health advocacy. Participants were able to apply these skills to identify and address health disparities, develop entrepreneurial solutions to health problems, and advocate for policy changes that support health innovations. Lastly, Backes (2022) provided a comprehensive evaluation of skill development, focusing on self-empowerment, adaptability, and innovative practices. The study showed that participants acquired skills in adapting to changing health environments, implementing innovative health solutions, and empowering themselves and others to take initiative in health innovation projects.

**Evaluation of social innovation in health knowledge**

Only four manuscripts (20%) evaluated social innovation in health knowledge (Gilmartin, 2013; Ezezika 2021; Backes, 2022; VanNiekerk 2022). We observed that the common outcomes across these manuscripts when evaluated were twofold: first, they highlighted that comprehensive knowledge development—both theoretical and practical—was crucial for effectively driving health innovation. Participants consistently demonstrated the ability to apply this knowledge to real-world health challenges, thereby fostering innovative solutions and advocating for policy changes. Second, the manuscripts underscored the importance of experiential learning methods, such as workshops, course assignments, and participatory research, which significantly enhanced participants' knowledge and ability to implement health innovations. These findings collectively illustrate that a diverse set of evaluation methods—surveys, course assignments, observational techniques, and participatory action research—is instrumental in building capacity by enriching both theoretical understanding and practical application. This approach is vital for equipping individuals who are interested in social innovation in health to meet the evolving challenges in healthcare with effective and innovative solutions.

For instance, Gilmartin (2013) used course assignments and surveys to evaluate improvements in participants' understanding of social disparities and their ability to identify opportunities for social entrepreneurship and public health leadership. The study revealed significant gains in knowledge, empowering participants to develop innovative solutions to health disparities and advocate for necessary policy changes. Similarly, Ezezika (2021) employed a combination of surveys and observational methods to assess the process of preparing and delivering entrepreneurial pitches. The outcomes indicated a substantial enhancement in knowledge related to health entrepreneurship and innovation, providing participants with practical insights into translating innovative ideas into impactful health interventions. In another approach, Backes (2022) utilized surveys and participatory action research to evaluate the depth of knowledge regarding the implementation of innovative health solutions. The study demonstrated that participants developed a comprehensive understanding of health systems and innovation strategies, which were essential for addressing complex health challenges and fostering sustainable solutions. Furthermore, Van Niekerk (2022) relied on workshops and webinars as platforms for knowledge exchange among stakeholders. This study focused on practical knowledge transfer and co-learning, highlighting a deep understanding of stakeholder dynamics and the critical role of collaboration in sustaining health innovation.

These manuscripts collectively demonstrate that diverse evaluation methods—such as surveys, course assignments, observational techniques, and participatory action research—are essential for enhancing both theoretical and practical knowledge. This comprehensive approach equips individuals to respond effectively to the dynamic challenges in healthcare, driving sustainable and innovative solutions.
