# Supplementary material for: Global learning opportunities within social innovation in health (GLOWS): A modified Delphi process to identify and pilot core competencies for learning
Source: PLoS One. 2026 Jan 9;21(1):e0339359. doi: 10.1371/journal.pone.0339359 (PMC12788671; doi:10.1371/journal.pone.0339359)
Supplement: S9 File — (DOCX) [file pone.0339359.s009.docx]

Social Innovation in Health Mid-Year Training Workshop - Post-training Survey

Start of Block: Default Question Block

Thank you for participating in the Social Innovation in Health Mid-Year Training Workshop. Your feedback is important for us to improve future training workshops. All data collected will be kept strictly confidential and not shared with others. Thank you for your time!

Demographics and Background

Q1 Q1. What is your first and last name?

________________________________________________________________

| 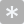 |
| --- |

Q2 Q2. What is your email address?

________________________________________________________________

Q3 Q3. What is your age in years?

________________________________________________________________

Q4 Q4. How do you identify your gender?

- Male (1)
- Female (2)
- Non-binary / third gender (3)
- Prefer not to say (4)

Q5 Q5. According to your national definitions, are you a member of a racial or ethnic minority?

- A. Yes (1)
- B. No (2)
- C. Prefer not to say (3)

Q6 Q6. Which country do you live in?

________________________________________________________________

Q7 Q7. Are you currently a student (undergraduate, graduate, or postgraduate)?

- Yes (1)
- No (2)

Q8 Q8. What is the highest level of education you have attained?

- A. High school (1)
- B. Bachelors (2)
- C. Masters (3)
- D. Ph.D. or MD (4)

Q9 Q9. What is your main field of study/expertise?

________________________________________________________________

Q10 Q10. What type of research are you interested in? (open response)

________________________________________________________________

________________________________________________________________

________________________________________________________________

________________________________________________________________

________________________________________________________________

| Page Break |  |
| --- | --- |

Display this question:

If Q7. Are you currently a student (undergraduate, graduate, or postgraduate)? = No

Q11 Q11. What is your primary job duty?

- A. Research (1)
- B. Teaching (2)
- C. Service (3)
- D. Policy (4)
- E. Clinician (5)
- F. Other (6)

Display this question:

If Q7. Are you currently a student (undergraduate, graduate, or postgraduate)? = No

Q12 Q12. How long have you worked in this field (years)?

________________________________________________________________

Display this question:

If Q7. Are you currently a student (undergraduate, graduate, or postgraduate)? = No

Q13 Q13. What is your Position/Title?

________________________________________________________________

Display this question:

If Q7. Are you currently a student (undergraduate, graduate, or postgraduate)? = No

Q14 Q14. What is the name of your Institution/Workplace?

________________________________________________________________

| Page Break |  |
| --- | --- |

Workshop Experience and Outcomes

Q15 Q15. How many of the eight sessions did you attend or watch online? (check all that apply)

- A. Introduction to Participatory Health Research Methods Emphasizing Designathons (1)
- B. Social Innovation in Health: Introduction to Social Innovation Theory and KAP-B Framework (2)
- C. Introduction to Human-Centered Design for Health (3)
- D. Applying an Intersectional Gender Lens on Social Innovations in Health: An Introductory Module (4)
- E. Co-Creation: An Innovative Communal Approach in Addressing Social Health Challenges (5)
- F. Democratizing Applied Behavioral and Decision Sciences to Combat MMR Vaccine Hesitancy through Behavioral Design Sprints (6)
- G. Embedding Social Innovation to Strengthen Health Systems and Leadership (7)
- H. From Idea to Impact: Mastering the Pitch for Social Innovations (8)

Q16 Q16. On a scale of 1-5, please rate your competencies in social innovation in health for each of the following aspects after the workshop where 1 indicates "Not Competent at All" and 5 indicates "Very Competent":

|  | 1 (1) | 2 (2) | 3 (3) | 4 (4) | 5 (5) |
| --- | --- | --- | --- | --- | --- |
| Understanding health disparities and their roots in intersectional inequities (1) |  |  |  |  |  |
| Building empathy and the ability to deeply connect with local communities of interest (2) |  |  |  |  |  |
| Developing leadership skills to nurture relationships with local communities, enhance multi-stakeholder partnerships, and manage diverse groups (3) |  |  |  |  |  |
| Cultivating a growth mindset to manage and expect failures, learn over time, and develop resiliency (4) |  |  |  |  |  |
| Practicing adaptability to rapidly iterate and respond to local contexts and feedback (5) |  |  |  |  |  |
| Enhancing communication skills to effectively communicate with a broad range of communities, especially people with lived experience and potential partners (6) |  |  |  |  |  |
| Strengthening entrepreneurial skills to raise funds for social innovation, develop innovative financing and sustainability approaches, and rapidly iterate ideas (7) |  |  |  |  |  |

Q17 Q17. How would you rate your knowledge of social innovation in health after the workshop?

- A. Very knowledgeable (1)
- B. Knowledgeable (2)
- C. Somewhat knowledgeable (3)
- D. Slightly knowledgeable (4)
- E. Not knowledgeable at all (5)

Q18 Q18. How confident are you in applying social innovation concepts to your work after the workshop?

- A. Very confident (1)
- B. Quite confident (2)
- C. Moderately confident (3)
- D. Slightly confident (4)
- E. Not confident at all (5)

Q19 Q19. To what extent were your goals met during the workshop?

- A. Completely met (1)
- B. Mostly met (2)
- C. Partially met (3)
- D. Slightly met (4)
- E. Not met at all (5)

Q20 Q20. Please rate your satisfaction with the following workshop topics on a scale from 1 to 5 where 1 indicates "Not Satisfied" and 5 indicates "Very Satisfied":

|  | 1 (1) | 2 (2) | 3 (3) | 4 (4) | 5 (5) |
| --- | --- | --- | --- | --- | --- |
| Introduction to Participatory Health Research Methods Emphasizing Designathons (1) |  |  |  |  |  |
| Social Innovation in Health: Introduction to Social Innovation Theory and KAP-B Framework (2) |  |  |  |  |  |
| Introduction to Human-Centered Design for Health (3) |  |  |  |  |  |
| Applying an Intersectional Gender Lens on Social Innovations in Health (4) |  |  |  |  |  |
| Co-Creation: An Innovative Communal Approach in Addressing Social Health Challenges (5) |  |  |  |  |  |
| Democratizing Applied Behavioral and Decision Sciences to Combat MMR Vaccine Hesitancy (6) |  |  |  |  |  |
| Embedding Social Innovation to Strengthen Health Systems and Leadership (7) |  |  |  |  |  |
| From Idea to Impact: Mastering the Pitch for Social Innovations (8) |  |  |  |  |  |

Q21 Q21. Overall, how would you rate the overall organization and delivery of the workshop?

- A. Excellent (1)
- B. Good (2)
- C. Fair (3)
- D. Poor (4)

| Page Break |  |
| --- | --- |

Additional Information

Q22 Q22. Are you associated with a SIHI Hub? If yes, list here:

________________________________________________________________

________________________________________________________________

________________________________________________________________

________________________________________________________________

________________________________________________________________

Q23 Q23. How did you find out about this training workshop? (check all that apply)

- A. Organization's website (1)
- B. Social media (2)
- C. Email (3)
- D. Referral (4)
- E. Other (please specify) (5) __________________________________________________

Q24 Q24. Would you attend our workshop in the future?

- A. Yes (1)
- B. No (2)

Q25 Q25. How likely are you to recommend this workshop to a colleague or friend?

- A. Very likely (1)
- B. Likely (2)
- C. Neutral (3)
- D. Unlikely (4)
- E. Very unlikely (5)

Q26 Q26. What were the most valuable aspects of the workshop for you? (open response)

________________________________________________________________

________________________________________________________________

________________________________________________________________

________________________________________________________________

________________________________________________________________

Q27 Q27. Were there any aspects of the workshop that you found less useful or that need improvement? (open response)

________________________________________________________________

________________________________________________________________

________________________________________________________________

________________________________________________________________

________________________________________________________________

Q28 Q28. What topics would you like to see covered in future workshops?

________________________________________________________________

Q29 Q29. Would you like to be added to our SESH email list to receive periodic updates on social innovation, research, and capacity building?（If yes, please provide your preferred email address）

- A. Yes (1) __________________________________________________
- B. No (2)

Q30 Q30. Our team is working to develop a consensus statement on social innovation learning competencies. Are you interested in contributing to the dissemination of the resulting statement? This would involve helping to develop infographics, videos, and other material relevant to the consensus. If you select yes, your contact details will be shared with the project organizing committee.

- A. Yes (1)
- B. No (2)

End of Block: Default Question Block
