# Supplementary material for: Global learning opportunities within social innovation in health (GLOWS): A modified Delphi process to identify and pilot core competencies for learning
Source: PLoS One. 2026 Jan 9;21(1):e0339359. doi: 10.1371/journal.pone.0339359 (PMC12788671; doi:10.1371/journal.pone.0339359)
Supplement: Attachment — Submitted filename: review R3_ manuscript_PlosOne.pdf [file pone.0339359.s013.pdf]

Dear Authors,

I have completed my review of the revised manuscript and the authors' responses to previous reviewer comments. The authors have made substantial improvements to the manuscript, demonstrating careful consideration of feedback and meaningful revisions that enhance the overall quality and clarity of this important work.

However, two specific areas require minor clarification to fully optimize the manuscript's impact and ensure complete alignment with journal publication standards:

1. Abstract. The statement *"After the workshops, 137 participants completed a survey rating their competency on a scale from 1 (not competent) to 5 (very competent) most reported a significant 1-point improvement"* lacks specificity regarding which competency areas showed improvement. Specify the particular competency domains where participants reported improvement. This specification will strengthen the abstract's informativeness and help readers better understand the workshop outcomes.
2. Suggestion: create a separate "Conclusion" section that incorporates and expands upon the final paragraph of the current Discussion section. This section should briefly provide: a synthesis of the study's main findings; an outline of the major contributions to social innovation in health education, implications for research and practice, and suggested directions for future research.

**Recommendation**

The manuscript represents a valuable contribution to social innovation in health education and is nearly ready for publication. I recommend <u>minor revision</u> with specific attention to the issues outlined above. These are straightforward modifications that should not require extensive rewriting. Once these minor issues are addressed, the manuscript will meet the high standards expected for PLOS ONE publication.
