# Supplementary material for: Global learning opportunities within social innovation in health (GLOWS): A modified Delphi process to identify and pilot core competencies for learning
Source: PLoS One. 2026 Jan 9;21(1):e0339359. doi: 10.1371/journal.pone.0339359 (PMC12788671; doi:10.1371/journal.pone.0339359)
Supplement: Attachment — Submitted filename: Review manuscript PlosOne.docx [file pone.0339359.s010.docx]

Dear Authors,

Thank you for giving me the opportunity to review your work.

This study employs a modified Delphi method to identify and establish a consensus on 30 core learning competencies in the field of social innovation in health, which are categorized into skills, mindsets and knowledge. Eight online workshops were conducted to pilot 12 of those 30 competencies. The results of this study are anticipated to contribute to the development of a WHO/TDR^[1]^ conceptual framework and to inform the standardization of social innovation in health education and training.

The topic is timely and attempts to address a methodological gap in the integration of social innovation into health education. The use of a modified Delphi method — engaging diverse and heterogeneous panel of social innovation experts from low-, middle-, and high-income countries — ensures the competencies reflect real-world needs across varied contexts, thereby enhancing their relevance and applicability. The focus on low-resource contexts addresses an empirical critical gap in social innovation research, which has historically been centred on high-income settings. Furthermore, as the study is conducted under the auspices of the WHO/TDR program, the findings are expected to directly inform policy and institutional strategies, provinding a roadmap for the implementation of standardized training worldwide.

Despite its merits, the manuscript requires major revisions to improve transparency and clarity. Having read the manuscript two times, I present below my concerns/critiques/suggestions:

**Major issues/Major concerns**

1. The scoping review (ScR) currently included as Supplementary Material plays a fundamental role in the development the Delphi process and conceptual framework. I recommend incorporating it within the main manuscript (after addressing the specific scoping review comments identified below) to allow readers to fully understand this critical methodological component and its influence on subsequent phases of your research.
2. Introduction. While the Introduction identifies gaps in social innovation education (e.g., inconsistency in competencies and resource disparities between high- and low-income settings), it would benefit from a stronger foundation on the concept of social innovation itself, its application in health contexts, and its positioning within educational frameworks. This additional context would help situate your study within the broader scholarship in these intersecting fields.
3. Introduction. I encourage you to elaborate on the broader implications of standardizing competencies. While you mention the study will "inform training programmes," expanding on how this standardization could influence policy, scale innovations, or reduce health disparities (particularly in LMIC contexts) would enhance the manuscript's relevance. Consider connecting your research to potential outcomes such as workforce development and community empowerment.
4. Introduction. Your grouping of competencies into knowledge, skills, and mindset categories would benefit from theoretical justification. For instance, the KSA (Knowledge-Skills-Attitudes) framework proposed by Benjamin Bloom ([Bloom et al., 1956](https://www.sciencedirect.com/science/article/pii/S1471595324000623#bib10)), which is widely recognized across education, and human resource training literature, could be employed.
5. Introduction. The transition from problem identification to study objectives appears somewhat abrupt. Consider reorganizing to create a clearer connection between the identified gaps (e.g., fragmented LMIC training resources) and your study's specific aims. Framing the study as a response to WHO/TDR's call for action would enhance narrative cohesion.
6. Introduction. While your introduction highlights the importance of social innovation in global health education, explicit research questions would strengthen the manuscript. I recommend adding a concise paragraph stating your specific research questions, demonstrating how they align with the identified gaps, and outlining how you intend to address them.
7. Methods. Please clarify how the scoping review and crowdsourcing open call specifically informed the Delphi process design. An overview figure linking each methodological step would provide valuable clarity on how these components connect and build upon each other.
8. Methods. The relatively low participation rate in the first Delphi round (67.6%) and further attrition in Round 2 raise potential bias concerns. I recommend discussing steps taken to mitigate attrition and acknowledging its potential impact on consensus validity.
9. Methods. The rationale for piloting 12 out of 30 competencies requires clearer explanation. Please revise this section to detail why these specific competencies were selected for piloting over others.
10. Discussion. The geographic imbalance in participant recruitment, particularly the underrepresentation of low-income regions, deserves discussion as a limitation potentially affecting the framework's universal applicability.
11. Discussion. While attrition in the Delphi process is noted, reflecting on how participant dropouts might have influenced consensus would enhance methodological transparency.

**Minor issues/Minor comments**

1. Line 36: Specify the exact number of competencies piloted (12) to avoid ambiguity.
2. Introduction. The choice of a modified Delphi method warrants more thorough justification. I suggest explaining why this method was selected over alternatives (e.g., nominal group technique) and how it specifically aligns with your study's participatory goals. A brief discussion of the Delphi method's particular strengths in your context would strengthen methodological rigor.
3. Introduction. The frequent use of institutional acronyms (SIHI, TDR, SESH) may reduce clarity for readers unfamiliar with these organizations/initiatives. Consider adding a "Study Context" section that defines each institution's mandate and relevance to your research. This would help readers understand how these partnerships enhanced your study design, recruitment approach, and policy implications.
4. Line 510: Renumber the table summarizing learning competency statements, as there are currently two tables designated as Table 1.
5. Results. A table with demographic information of the Delphi panel participants in both rounds - included in the manuscript text - would enhance methodological transparency and allow readers to assess the diversity and representativeness of your expert panel.
6. Results. Figure 3 requires clarification. The y-axis scale should be clearly defined, and the caption "B. confidence in applying these concepts post-workshop" is confusing since the figure displays both pre- and post-intervention results. Consider revising for greater clarity.
7. Results. Consider including the table with Delphi Round 2 results within the main manuscript rather than supplementary materials.
8. Discussion. While the pilot workshops demonstrated improved self-reported competencies, I suggest acknowledging the limitation of not including objective metrics (e.g., post-intervention behavioural changes or number of projects implemented) to evaluate real-world impact.
9. Discussion. To strengthen policy relevance, I recommend expanding on how institutions like WHO/TDR might operationalize these competencies, perhaps through accreditation systems or funding priorities.
10. Discussion. Consider discussing how the identified competencies might be adapted to local contexts or scaled within existing health systems—critical considerations for policymakers and educators seeking to implement your framework.
11. Discussion/Future Research: To enhance impact, I suggest proposing concrete steps for operationalizing the competencies (e.g., integration into national curricula, partnerships with local NGOs) and acknowledging the need for future research to evaluate whether training translates to sustained innovation or equity improvements.

**Review of the document reporting the scoping review and crowdsourcing open call (document presented as Supplementary Material):**

**Summary:**

This document presents a comprehensive evidence synthesis that combines a scoping review and a crowdsourcing open call to identify competencies and teaching strategies for social innovation in health. The authors identified 16 core competencies categorized into skills, mindsets, and knowledge domains, with particular emphasis on seven key competencies: problem-solving skills, communication skills, creative thinking, collaborative mindsets, community-engaged participatory methods, health disparity knowledge, and intersectionality.

The mixed-methods approach for collecting and synthesizing evidence is innovative and helps the addresses on of the authors’ identified research gaps in the literature: the lack of literature considering the realities of LMIC contexts. Indeed, the deliberate effort to include perspectives from LMICs through the open call (with 89% of eligible entries from LMICs) helps address the bias in the literature toward high-income settings.

Also, registered their protocol on Open Science Framework (OSF), enhancing the transparency and replicability of their methods.

Nevertheless, this document requires significant revisions to enhance its methodological rigor, transparency, and overall clarity. As previously mentioned, I strongly recommend incorporating the scoping review and crowdsourcing open call—including the complete methodological protocol and results—into the main manuscript rather than presenting them as supplementary material. This integration would provide readers with a more comprehensive understanding of your foundational work. Below, I present my specific concerns and suggestions for improvement:

**Major issues/Major concerns**

1. Introduction. The text would benefit from more comprehensive grounding with appropriate references regarding social innovation, its application to global health contexts, and its role in health education and training. This would help readers understand the scope and boundaries of social innovation in health and the importance of educational initiatives, particularly in LMICs.
2. Introduction. Please clearly state the specific research question(s) your scoping review addresses, ensuring alignment with your stated objectives.
3. Introduction. The text would benefit from a clearer explanation of how the scoping review informs subsequent research steps. I recommend rewriting this section.
4. Methods. Lines 87-91. Please specify (using authors' initials) which two researchers performed the records screening and which third researcher resolved conflicts to enhance methodological transparency.
5. Methods. Your data analysis of both scoping review studies and open call documents would benefit from employing a robust qualitative research framework (e.g., content analysis, thematic analysis, or grounded theory).
6. Methods. I missed how the competencies identified in the scoping review studies were mapped in the textual analysis of the open call documents. Please explain how competencies identified in the scoping review were mapped during textual analysis of open call documents. Moreover, the current description of "descriptive analysis" lacks specific analytical procedures.
7. Methods. Include your criteria for selecting independent judges and explain how collective agreement among judges to select submissions was ensured.
8. Results. Consider reorganizing the Results section to more clearly distinguish findings from the scoping review versus the crowdsourcing open call.
9. Results. The study would benefit from a table synthesizing all reported competencies and their respective frequencies.
10. Discussion. The Discussion section should more thoroughly explore how competencies might vary across different contexts. While you mention conducting subgroup analysis comparing high-income and LMIC settings, the results and implications of these similarities/differences warrant more detailed discussion.
11. Figure 1. In Figure 1, please specify what constitutes the "N=20 reports of included studies" in addition to the N=20 studies included in the review.
12. Figure 3. While visually appealing, this figure needs additional explanation. Please specify that it is a bubble chart and explain the significance of the different circle sizes and colours.

**Minor issues/minor concerns**

1. Introduction. I recommend providing a clearer explanation of what constitutes a crowdsourcing open call and how it differs from other participatory approaches. Including references to other studies that have utilized this approach would be helpful for the reader to situate this study within the broader literature.
2. Methods. The statement on line 43 regarding "limitations in the amount and geographic scope of existing studies (…)" would be more appropriate in the Discussion section rather than Methods.
3. Methods. Line 50. Please correct the reference for the Joanna Briggs Institute (JBI). The primary reference should be: Peters MD, Godfrey CM, Khalil H, McInerney P, Parker D, Soares CB. Guidance for conducting systematic scoping reviews. Int J Evid Based Healthc. 2015;13(3):141-6.
4. Methods. Include your search strategies for each electronic database as Supplementary Material for transparency and reproducibility.
5. Methods. Line 86. Include an appropriate reference for the Covidence software.
6. Results. Including your data extraction form as Supplementary Material would enhance methodological transparency.
7. Results. Provide a full list of the references included in your scoping review as Supplementary Material.
8. Results. Consider creating a table with general characteristics of each included study to improve clarity and organization of findings.
9. Results. A detailed table of descriptive/methodological characteristics would be helpful, separating studies reporting competencies from those reporting teaching methods and strategies.
10. Results. The balance between reporting results from the scoping review versus the open call appears uneven. Providing more details on the evaluation of open call submission documents would strengthen this section.

1. *WHO/TDR, the Special Programme for Research and Training in Tropical Diseases, is a global programme of scientific collaboration that helps facilitate, support and influence efforts to combat diseases of poverty. It is co-sponsored by the United Nations Children’s Fund (UNICEF), the United Nations Development Programme (UNDP), the World Bank and the World Health Organization (WHO).
