# Supplementary material for: Global learning opportunities within social innovation in health (GLOWS): A modified Delphi process to identify and pilot core competencies for learning
Source: PLoS One. 2026 Jan 9;21(1):e0339359. doi: 10.1371/journal.pone.0339359 (PMC12788671; doi:10.1371/journal.pone.0339359)
Supplement: Attachment — Submitted filename: Response to Reviewers.docx [file pone.0339359.s011.docx]

Manuscript PONE-D-25-16004

Global Learning Opportunities Within Social Innovation in Health (GLOWS): Modified Delphi Process to Identify and Pilot Core Competencies for Learning

Dear Prof Monica Duarte Correia de Oliveira,

Thank you for the helpful comments on this manuscript. We have used the comments to strengthen the manuscript. Find below point-by-point responses.

Thank you for your consideration.

Sincerely,

Emily and Joe on behalf of the co-authors

**UPDATED FUNDING STATEMENT:** The work received support from TDR, the Special Programme for Research and Training in Tropical Diseases co-sponsored by UNICEF, UNDP, the World Bank and WHO. TDR is able to conduct its work thanks to the commitment and support from a variety of funders. These include our long-term core contributors from national governments and international institutions, as well as designated funding for specific projects within our current priorities. For the full list of TDR donors, please visit TDR’s website at: <https://www.who.int/tdr/about/funding/en/> TDR receives additional funding from Sida, the Swedish International Development Cooperation Agency, to support SIHI.

**UPDATED COMPETING INTERESTS STATEMENT:** The authors declare the following potential conflicts of interest: Angelica Borbón, Bertha M. Chakhame, Adriana S. Ruiz, Abigail Ruth Mier, Obidimma Ezezika, Jana Deborah Mier-Alpano, and Gifty Marley have received honoraria for speaking at the pilot workshops. These payments were provided by SESH, the SIHI Hub in China. They declare no other potential conflicts of interest. This does not alter our adherence to PLOS ONE policies on sharing data and materials.

**COMMENT 1: The scoping review (ScR) currently included as Supplementary Material plays a fundamental role in the development the Delphi process and conceptual framework. I recommend incorporating it within the main manuscript (after addressing the specific scoping review comments identified below) to allow readers to fully understand this critical methodological component and its influence on subsequent phases of your research.**

RESPONSE 1: We agree that the scoping review plays a fundamental role in the Delphi process. The scoping review and open call are detailed in a separate related paper, which is currently under peer-review with another journal. We included it as supplementary material because the pre-print was not available. We have now referenced the pre-print. This does not constitute dual publication, as the scoping review and the open call employed distinct methodologies to this paper. Additionally, it is beyond the scope of a single paper to provide a detailed account of the open call and scoping review alongside the modified Delphi process described in this manuscript.

We have also elaborated on the scoping review and open call in this paper as advised providing further details on the methodology and how it informed the Delphi process and pilot workshops.

**COMMENT 2: Introduction. While the Introduction identifies gaps in social innovation education (e.g., inconsistency in competencies and resource disparities between high- and low-income settings), it would benefit from a stronger foundation on the concept of social innovation itself, its application in health contexts, and its positioning within educational frameworks. This additional context would help situate your study within the broader scholarship in these intersecting fields.**

RESPONSE 2: We agree that providing additional context would benefit this paper. We have expanded the introduction section to reflect these key points.

**COMMENT 3: Introduction. I encourage you to elaborate on the broader implications of standardizing competencies. While you mention the study will "inform training programmes," expanding on how this standardization could influence policy, scale innovations, or reduce health disparities (particularly in LMIC contexts) would enhance the manuscript's relevance. Consider connecting your research to potential outcomes such as workforce development and community empowerment.**

RESPONSE 3: We agree that there are broader implications to standardizing competencies beyond informing training programmes. We have elaborated on this in the updated manuscript to describe the benefits of aligning educational curricula including in LMIC contexts. Ultimately, this standardization could help to empower local populations to become active participants in co-producing health outcomes which could lead to reduced health disparities.

**COMMENT 4: Introduction. Your grouping of competencies into knowledge, skills, and mindset categories would benefit from theoretical justification. For instance, the KSA (Knowledge-Skills-Attitudes) framework proposed by Benjamin Bloom (****[Bloom et al., 1956](https://www.sciencedirect.com/science/article/pii/S1471595324000623#bib10)), which is widely recognized across education, and human resource training literature, could be employed.**

RESPONSE 4: We agree that justifying the grouping of competencies would strengthen the paper and we have included a reference to the KSA framework from Benjamin Bloom.

**COMMENT 5: Introduction. The transition from problem identification to study objectives appears somewhat abrupt. Consider reorganizing to create a clearer connection between the identified gaps (e.g., fragmented LMIC training resources) and your study's specific aims. Framing the study as a response to WHO/TDR's call for action would enhance narrative cohesion.**

RESPONSE 5: We agree that the transition was somewhat abrupt, and we have edited to ensure better flow between the identified gaps and the study’s specific aims.

**COMMENT 6: Introduction. While your introduction highlights the importance of social innovation in global health education, explicit research questions would strengthen the manuscript. I recommend adding a concise paragraph stating your specific research questions, demonstrating how they align with the identified gaps, and outlining how you intend to address them.**

RESPONSE 6: We agree that including explicit research questions would strengthen the manuscript hence we have included an additional paragraph at the end of the introduction to address this. It highlights that the study aims to identify the core competencies in social innovation in health and explore whether these competencies could be effectively taught and engaged with in a virtual setting.

**COMMENT 7: Methods. Please clarify how the scoping review and crowdsourcing open call specifically informed the Delphi process design. An overview figure linking each methodological step would provide valuable clarity on how these components connect and build upon each other.**

RESPONSE 7: We agree that elaborating on how the open call and scoping review informed the Delphi would be beneficial. To address this, we included further details on the open call and scoping review, including the number of submissions and studies included and that the themes extracted from both the open call and literature review were constructed into statements which were distributed in round one of the modified Delphi process.

**COMMENT 8: Methods. The relatively low participation rate in the first Delphi round (67.6%) and further attrition in Round 2 raise potential bias concerns. I recommend discussing steps taken to mitigate attrition and acknowledging its potential impact on consensus validity.**

RESPONSE 8: We agree that it is important to discuss steps which were taken to mitigate attrition and to acknowledge its potential impact. We have included further detail on this in the discussion section. We outline that participant dropout during the Delphi process may have influenced the consensus by reducing the diversity of perspectives and potentially skewing results toward the views of those who remained engaged. However, steps were taken to mitigate attrition, including sending reminder emails and coordinating multiple teleconferences.

**COMMENT 9: Methods. The rationale for piloting 12 out of 30 competencies requires clearer explanation. Please revise this section to detail why these specific competencies were selected for piloting over others.**

RESPOSNE 9: We agree that further clarity on why only 12/30 competencies were piloted would improve the paper. We have now described that only twelve out of thirty were piloted due to time and resource constraints as well as the areas of expertise of the facilitators. The workshops were co-designed by people who submitted the highest scoring open-call applications.

**COMMENT 10: Discussion. The geographic imbalance in participant recruitment, particularly the underrepresentation of low-income regions, deserves discussion as a limitation potentially affecting the framework's universal applicability.**

RESPONSE 10: Ensuring diverse representation was a priority for the research team in the recruitment of participants as well as in the study design. We have included further details on the representation from both LMICs and HICs which we believe strengthens the frameworks universal applicability. The fifth paragraph in the discussion section now describes that 22/46 (47.8%) of participants in the first survey were from LMICs. Several aspects of the study enabled this recruitment of such a diverse group. As with the recruitment process, both surveys and the teleconference were online, improving accessibility as participants were not required to travel. The scoping review and open call were also led by researchers from LMICs. While much of the literature that was analysed in the scoping review was produced in HICs, many of the open call submissions were from LMICs allowing for diverse experiences to be accounted for in the initial development of statements.

**COMMENT 11: Discussion. While attrition in the Delphi process is noted, reflecting on how participant dropouts might have influenced consensus would enhance methodological transparency.**

RESPONSE 11: We agree, and we have now described how participant dropout during the Delphi process may have influenced the consensus by reducing the diversity of perspectives and potentially skewing results toward the views of those who remained engaged in the discussion section. This has been revised in the discussion section.

**COMMENT FROM REVIEWER 2: First, embedding the study into the wider context of social innovation and health is missing, and relations of the study to previously published research is scarce. Second, key concepts of the paper, such as core competencies and social innovation are lacking clear definitions. Third, I do not understand the goal of the piloting and how it relates to the Delphi process. Fourth, the methodological process is difficult to follow. Fifth, the results are partly not aligned with the methodological steps. Sixth, the discussion section does not discuss all the results.**

RESPONSE: We agree that embedding the study into the wider context and better defining key concepts would benefit the paper and we have edited accordingly.

Regarding the third point, the goal of the piloting was to assess the feasibility and perceived value of delivering the competencies identified through the Delphi process in an online workshop format. While the Delphi process was used to reach consensus on the core competencies, the piloting phase aimed to explore whether these competencies could be effectively communicated and engaged with in a virtual setting. We have clarified this relationship in the revised manuscript.

In respect to the fourth point, we have revised figure one which depicts the methodological steps in for clarity.

Fifth, we have edited the manuscript to improve the alignment between the results and the methodological steps.

Finally, discussing each competency individually was outside the scope of this manuscript. We have highlighted the competencies which reached highest levels of consensus. We have strengthened the discussion section by elaborating on the learning resources, workshops and potential limitations.

**MINOR REVISIONS**

**On line 118 you refer to figure 1 but the correct figure is figure 2.**

RESPONSE: This is now corrected.

**Please insert a reference to table 1 in the first paragraph of the results section. The demographic details of the participants of round two should be provided to see the changes in the composition of the participants.**

RESPONSE: This is now included.

**In figure 1, please indicate that 34 participants joined the teleconferences sessions.**

RESPONSE: This is now included.

**On line 231 you write that one participant was excluded from the second survey. So, 35 participants were left but in figure 1 you indicate that 34 participants participated at the second survey.**

RESPONSE: This is now amended in figure 1**.**

**On line 241 you write that you identified 30 competencies but figure 2 lists less than 30 competencies.**

RESPONSE: To enhance clarity and readability, we chose not to include all 30 competencies in Figure 2, as doing so would have compromised the visual effectiveness of the graphic. Instead, we highlighted the competencies that received the highest levels of consensus and were considered most important.

**On line 289 you refer to table 2 but in the appendix it is table 1.**
RESPONSE: This is now corrected.

**In table 1 you mention the preamble as a theme. This is the first and only time the preamble is mentioned in the paper. I do not understand what it is. Also, the three bullet points of the preamble description are not competencies, in contrast to the bullet points in the description of the other themes.**

RESPONSE: We have updated the results section to give further details on the preamble. We describe that the preamble was an added item in the second survey as per feedback on the first edition. It contained the definition of social innovation, outlined the reasons for why training and education are important to achieve impactful innovation in health, the primary users of the consensus statement and different mechanisms to achieve competencies in social innovation.

**Line 36: Specify the exact number of competencies piloted (12) to avoid ambiguity.**

RESPONSE: We agree with this recommendation which now has been amended.

**Introduction. The choice of a modified Delphi method warrants more thorough justification. I suggest explaining why this method was selected over alternatives (e.g., nominal group technique) and how it specifically aligns with your study's participatory goals. A brief discussion of the Delphi method's particular strengths in your context would strengthen methodological rigor.**

RESPONSE: The beginning of the methods section now highlights why the modified Delphi was used and its advantages over other methods.

**Introduction. The frequent use of institutional acronyms (SIHI, TDR, SESH) may reduce clarity for readers unfamiliar with these organizations/initiatives. Consider adding a "Study Context" section that defines each institution's mandate and relevance to your research. This would help readers understand how these partnerships enhanced your study design, recruitment approach, and policy implications.**

RESPONSE: We agree, there is now a Study Context paragraph at the end of the background section.

**Line 510: Renumber the table summarizing learning competency statements, as there are currently two tables designated as Table 1.**

RESPONSE: Numbering of tables has now been corrected amended.

**Results. A table with demographic information of the Delphi panel participants in both rounds - included in the manuscript text - would enhance methodological transparency and allow readers to assess the diversity and representativeness of your expert panel.**

RESPONSE: We agree, this is now included.

**Results. Figure 3 requires clarification. The y-axis scale should be clearly defined, and the caption "B. confidence in applying these concepts post-workshop" is confusing since the figure displays both pre- and post-intervention results. Consider revising for greater clarity.**

RESPONSE: We agree, this is now revised

**Results. Consider including the table with Delphi Round 2 results within the main manuscript rather than supplementary materials.**

RESPONSE: We agree, this is now included as table 3.

**Discussion. While the pilot workshops demonstrated improved self-reported competencies, I suggest acknowledging the limitation of not including objective metrics (e.g., post-intervention behavioural changes or number of projects implemented) to evaluate real-world impact.**

RESPONSE: We agree, this is now included.

**Discussion. To strengthen policy relevance, I recommend expanding on how institutions like WHO/TDR might operationalize these competencies, perhaps through accreditation systems or funding priorities.**

RESPONSE: We agree and now have elaborated on how competencies could be operationalized in the discussion section.

**Discussion. Consider discussing how the identified competencies might be adapted to local contexts or scaled within existing health systems—critical considerations for policymakers and educators seeking to implement your framework.**

RESPONSE: We agree and have elaborated on this in the discussion section including around diversifying the languages resources are available in and prioritizing participatory methods.

**Discussion/Future Research: To enhance impact, I suggest proposing concrete steps for operationalizing the competencies (e.g., integration into national curricula, partnerships with local NGOs) and acknowledging the need for future research to evaluate whether training translates to sustained innovation or equity improvements.**

**RESPONSE:** We agree. There is a need for future evaluation on the translation of training into sustained innovation and we have now mentioned this. We have elaborated on how competencies could be operationalized in the discussion section.
