# Supplementary material for: Global learning opportunities within social innovation in health (GLOWS): A modified Delphi process to identify and pilot core competencies for learning
Source: PLoS One. 2026 Jan 9;21(1):e0339359. doi: 10.1371/journal.pone.0339359 (PMC12788671; doi:10.1371/journal.pone.0339359)
Supplement: Attachment — Submitted filename: Response_to_Reviewers_auresp_2.docx [file pone.0339359.s012.docx]

Manuscript PONE-D-25-16004

Global Learning Opportunities Within Social Innovation in Health (GLOWS): Modified Delphi Process to Identify and Pilot Core Competencies for Learning

Dear Prof Monica Duarte Correia de Oliveira,

Thank you for the helpful comments on this manuscript. We have used the comments to strengthen the manuscript. Find below point-by-point responses.

Thank you for your consideration.

Sincerely,

Emily and Joe on behalf of the co-authors

**COMMENT 1:** Abstract. I would encourage you to improve the abstract by incorporating key specific (quantitative) results that demonstrate the strength of your findings. For instance, rather than the general statement "Some competencies reached higher levels of agreement than others" (lines-27-28) consider providing concrete percentages or numbers of competencies that achieved consensus. This specificity would better communicate the robustness of your consensus-building process to readers and enhance the abstract's overall quality.

**RESPONSE 1: We agree that including quantitative results strengthens the abstract and have amended the manuscript accordingly to reflect this.**

**COMMENT 2:** Abstract. The statement regarding "(…) most participants reported a significant improvement across six competencies." would benefit from greater precision and transparency. I recommend clarifying the measurement instrument employed. Moreover, what constituted “significance”? This additional detail would strengthen the credibility of your findings and assist readers in better understanding on how workshop participants’ improved their knowledge and skills.

**RESPONSE 2: We have expanded on the statement regarding post-workshop self-reported competency among participants as much as possible within the confines of the abstract word count limitation.**

**COMMENT 3:** Abstract. While the final sentences of your abstract are appropriate, I believe it somewhat understates the significant contributions of your work. I would suggest more explicitly articulating the practical implications for curriculum and training program design, competency framework development, and policy implementation. This enhancement would better reflect the substantial value your research offers to the global health education community.

**RESPONSE 3: We agree and have elaborated on this briefly to stay within the abstract word count limit.**

**COMMENT 4:** Introduction. In lines 75 and subsequent statements about social innovation being "underrepresented in formal educational frameworks" and training programs lacking "clarity around core competencies," I recommend strengthening these assertions with robust literature support. Additional references would substantiate these important claims and provide readers with a stronger foundation for understanding the research gap your study addresses.

**RESPONSE 4: Additional references have been added to support these statements.**

**COMMENT 5**: Introduction. To enhance the manuscript's clarity and focus, I encourage you to include a dedicated paragraph that explicitly states your specific research questions. This addition would demonstrate how your objectives align with the identified gaps in the literature and clearly outline your intended approach to addressing these important issues.

**RESPONSE 5: The research questions are now explicitly highlighted in the final paragraph of the background section.**

**COMMENT 6:** Regarding the statement on lines 233-234 about piloting twelve of thirty competencies due to "time and resource constraints as well as the areas of expertise of the facilitators," it would be valuable to have additional clarification about the facilitators' specific areas of expertise, the number of facilitators involved, and a more detailed description of the workshop procedures. This information would provide valuable context for understanding the workshop implementation approach.

**RESPONSE 6:** **Thank you for this helpful suggestion. We have revised the Methods section to provide additional clarification on the structure and delivery of the workshop. The training series was led by finalists from a global open call, each of whom was paired with a mentor with relevant expertise in social innovation, implementation research, or community engagement. These mentors supported the finalists in reviewing and refining their training materials and co-presenting the sessions. We have also added a more detailed description of the workshop procedures.**

**COMMENT 7**: Methods. The workshop evaluation would be significantly strengthened by including more comprehensive details about the pre-post assessment instruments and their validation procedures. I recommend considering the inclusion of these guides/templates used in the assessment surveys as supplementary material, which would enhance methodological transparency and facilitate replication by other researchers.

**RESPONSE 7: We have now added more comprehensive details about the pre-post assessment instruments, including descriptions of their development and any relevant validation procedures, in the revised Methods section. Additionally, we have included the survey used in the assessment as supplementary material.**

**COMMENT 8**: Discussion. Given the important WHO/TDR context of this work, I had anticipated a more detailed discussion of how these competencies could be operationalized within both existing training infrastructures and newly developed programs. Expanding this discussion would provide valuable guidance for practitioners and policymakers seeking to implement your framework and would enhance the practical utility of your research.

**RESPONSE 8: Further details on the training infrastructures that these competencies may be operationalized in is now included in the discussion.**

**COMMENT 9:** After re-reading, the reference to figure 2 at the end of the Survey Development section does not fit in my opinion. I suggest no reference to any figure here.
**RESPONSE 9: This reference is now removed.**

**COMMENT 10:** Line 32: A spacing correction is needed between the words "twelve" and "learning" to ensure proper formatting.

**RESPONSE 10: This has been corrected.**

**COMMENT 11:** References. I noticed some inconsistencies in the citation format throughout the reference list. Please ensure uniform adherence to the journal's citation style guidelines to maintain professional presentation standards.
 **RESPONSE 11: References have now been updated.**

**COMMENT 12:** Please insert references for the statements in the second and third sentences of the Background section.

**RESPONSE 12: References are now added.**

**COMMENT 13:** The Study Context section ends with “TDR” -> perhaps you intended to mention the TDR as collaboration partner?

**RESPONSE 13: This was an error and has now been corrected.**
