# Supplementary material for: Global learning opportunities within social innovation in health (GLOWS): A modified Delphi process to identify and pilot core competencies for learning
Source: PLoS One. 2026 Jan 9;21(1):e0339359. doi: 10.1371/journal.pone.0339359 (PMC12788671; doi:10.1371/journal.pone.0339359)
Supplement: Attachment — Submitted filename: Response to Reviewers Letter PLOS One.docx [file pone.0339359.s014.docx]

Manuscript PONE-D-25-16004

Global Learning Opportunities Within Social Innovation in Health (GLOWS): Modified Delphi Process to Identify and Pilot Core Competencies for Learning

Dear Prof Monica Duarte Correia de Oliveira,

Thank you for the helpful comment on this manuscript. We have used the comment to strengthen the manuscript. Find below response.

Thank you for your consideration.

Sincerely,

Emily and Joe on behalf of the co-authors

**COMMENT 1: In the Discussion section, on line 457, you write “There are several strengths to this study including diverse representation”. Following this topic sentence, the readers expect several strengths being discussed, however you only discuss diverse representation. I suggest to label diverse representation as the main strength of your paper (if you agree, of course) and say why it is of such high relevance to have diverse representation.**

RESPONSE 1: Thank you for your helpful comment. We have amended the manuscript to reflect this primary strength and we have expanded on why this diversity is important to ensure broad translation of the results.

**COMMENT 2. Abstract. The statement “After the workshops, 137 participants completed a survey rating their competency on a scale from 1 (not competent) to 5 (very competent) most reported a significant 1-point improvement” lacks specificity regarding which competency areas showed improvement. Specify the particular competency domains where participants reported improvement. This specification will strengthen the abstract's informativeness and help readers better understand the workshop outcomes.**

RESPONSE 2: Thank you for your comment. We have now included specific examples of competency domains in the abstract.

**COMMENT 3: create a separate "Conclusion" section that incorporates and expands upon the final paragraph of the current Discussion section. This section should briefly provide: a synthesis of the study's main findings; an outline of the major contributions to social innovation in health education, implications for research and practice, and suggested directions for future research.**

REPONSE 3: Thank you for your comment. We have now created a separate Conclusion section to address this.

**COMMENT 4: 1. Please ensure that you refer to Table 3 in your text as, if accepted, production will need this reference to link the reader to the Table.**

RESPONSE 4: This has now been corrected.
